# Mechanisms of Action of AGuIX as a Pan-Cancer Nano-Radiosensitizer: A Comprehensive Review

**DOI:** 10.3390/ph18040519

**Published:** 2025-04-02

**Authors:** Clémentine Aubrun, Tristan Doussineau, Léna Carmès, Aurélien Meyzaud, Fabien Boux, Sandrine Dufort, Adeline Delfour, Olivier De Beaumont, Céline Mirjolet, Géraldine Le Duc

**Affiliations:** 1 NH TherAguix SA, 19 Chemin des Prés, 38240 Meylan, France; doussineau@nhtheraguix.com (T.D.); carmes@nhtheraguix.com (L.C.); meyzaud@nhtheraguix.com (A.M.); fabien.boux@gmail.com (F.B.); dufort@nhtheraguix.com (S.D.); delfour@nhtheraguix.com (A.D.); olivier.de-beaumont@provepharm.com (O.D.B.); 2 X-Rain: Research Unit in Radiotherapy Combined with Immunotherapies and Nanoparticles, IMATHERA, Radiation Therapy Department, Centre Georges-François Leclerc, 21000 Dijon, France; cmirjolet@cgfl.fr; 3 TIReCS Team, CTM (Center for Translational and Molecular Medicine), INSERM UMR 1231, 21000 Dijon, France

**Keywords:** nanoparticle, radiosensitizer, radiotherapy, theranostic, nanomedicine, radiobiology, radioenhancement, ferroptosis, immune response, enhanced permeability and retention (EPR)

## Abstract

**Objective:** This review provides an overview of the current knowledge regarding the mechanisms of action of AGuIX, a clinical-stage theranostic nano-radiosensitizer composed of gadolinium. It covers the steps following the administration, from the internalization in tumor cells to the interaction with X-rays and the subsequent physical, chemical, biological, and immunological events. **Results:** After intravenous injection, AGuIX accumulates in tumors through the enhanced permeability and retention (EPR) effect, and its specific retention properties allow its persistence in tumors for several days. At the cellular level, the nanomedicine is internalized by endocytic processes and mainly located in the cytoplasm, especially in lysosomes. AGuIX enhances the effects of radiotherapy (RT) at several levels, starting from radiation–matter interactions to a chemical stage of reactive oxygen species (ROS) production, followed by a cascade of biological events leading to tumor cell death and immune response. Indeed, AGuIX induces a local increase in radiation dose deposition through the emission of Auger electrons, leading to a subsequent increase in ROS generation. AGuIX also impacts RT-induced biological mechanisms, including DNA damage and cell death mechanisms such as apoptosis, autophagic cell death, and ferroptosis. Last, the combination of AGuIX and RT stimulates an antitumor immune response through the induction of immunogenic cell death (ICD), the activation of dendritic and T cells, and the reprogramming of tumor-associated macrophages (TAMs) into a pro-inflammatory phenotype. **Conclusions:** AGuIX is a clinical-stage nanoparticle (NP) intravenously administered with pan-cancer potential due to its specific biodistribution properties and a strong ability to amplify RT-induced mechanisms.

## 1. Introduction

Cancer is one of the leading causes of death worldwide, with an estimation from the World Health Organization (WHO) of 10 million deaths in 2020 [1]. Radiation therapy, or radiotherapy (RT), is one of the three major modalities of cancer treatment, together with surgery and systemic treatments, including chemotherapy, targeted therapy, and immunotherapy. According to the WHO, more than 50% of cancer patients receive RT, and this number steadily increases each year as combination treatments are more and more applied. Basically, RT is a local cancer treatment consisting in the deposition of a lethal dose of ionizing radiation to the tumor that subsequently triggers cancer cell death. It could address both primary and metastatic tumors, and be administered as stand-alone therapy or in combination with other treatments, including surgery, and systemic therapies [2].

RT can be delivered by an external beam in external beam radiation therapy (EBRT), and administered with several types of radiation, including photons (X-rays and γ-rays), electrons, ions, and hadrons [3]. EBRT is commonly delivered by linear particle accelerators (LINACs) that generate X-rays because of their compacity. Indeed, charged particles and neutrons still require very specific installations, such as synchrotron facilities that limit their clinical use. RT can also be delivered from a radioactive agent placed inside the body [4]. This so-called internal RT can be administered either locally through the implantation of a radioactive material close to the tumor, as in brachytherapy, or systemically, through the delivery of a radiolabeled molecule. In systemic approaches, the radiopharmaceutical may target cancer cells by binding to specific receptors in targeted radionuclide therapy (TRT) or involvement in a specific metabolic pathway or by using the radionuclide’s natural affinity. To date, two beta emitters have been clinically approved for TRT (^177^Lu-PSMA-617 and ^177^Lu-DOTATATE), but other radiopharmaceutical-emitting beta and alpha particles, as well as Auger electrons, are also investigated [5]. Standards of care (SOCs) based on this approach are defined according to the specifications of the emitted species, i.e., half-life, energy, and linear energy transfer (LET). Moreover, they strongly depend on the biodistribution of the agent in the body and the tumor itself.

The main limitations of RT are the radiation toxicity to surrounding normal tissue that impairs the maximal dose that could be delivered and the tumor radioresistance [2]. Indeed, tumor control remains insufficient in most cases, and innovative radiation strategies are increasingly being studied to improve RT therapeutic index. In addition to TRT, new modalities are emerging, such as FLASH RT, which delivers ultra-high-dose rates (>40 Gy/s) of external radiation [6], and boron neutron capture therapy (BNCT) that activates in situ a ^10^B-containing agent through neutron irradiation [7]. Regarding conventional EBRT with photons, numerous progresses have been made through the years, including improvements of RT techniques, which allow for the modulation of intensity in time and space [8], furthermore supported by advances in refined dosimetry procedures [9], image-guidance [10], and adaptive RT [11]. These progresses benefit clinical practice with better planning and conformity of the administered radiation dose, thus refining the therapeutic window. However, the challenge is still to find a compromise between delivering a sufficient dose to control tumor growth and avoiding an increase in undesirable side effects or any damage to organs at risk (OARs) [12].

The differential response between tumor and normal is related to the 6 Rs of radiobiology, namely repair, redistribution, repopulation, reoxygenation, intrinsic radiosensitivity, and reactivation of antitumor immune response [13]. These guidelines translate most of the molecular processes that usually occur during and following radiation therapy. Repair refers to the reduced capacity of cancer cells to repair DNA damage induced by ionizing radiation. Redistribution is associated with changes in the cancer cell distribution in the different phases of the cell cycle, presenting significant differences in radiosensitivity. Repopulation is the phenomenon of tumor cell proliferation after exposure to ionizing radiation. Reoxygenation is a strategy mainly linked to fractionated RT regimens that exploit the capacity of hypoxic cancer cells, more radioresistant than normoxic ones, to reoxygenate between RT fractions. Intrinsic radiosensitivity refers to the capacity of certain therapeutic agents to inhibit specific genetic targets responsible for radioresistance. Finally, reactivation of the immune response is related to the ability of RT to modify the tumor microenvironment (TME) and to activate both innate and adaptive immunity.

Ionizing radiation can cause tumor cell death by inducing DNA damage, either directly or indirectly, with the latter accounting for 60 to 99% of the effects depending on the context [14,15,16]. Indeed, incoming photons could directly ionize DNA, inducing single-strand breaks (SSBs) and double-strand breaks (DSBs), potentially leading to tumor cell apoptosis or necrosis. On the other hand, the indirect process starts with water radiolysis, i.e., photon absorption by water molecules, resulting in the generation of reactive oxygen species (ROS), in particular, hydroxyl radical (OH^•^), hydrogen peroxide (H_2_O_2_), and superoxide ion (O_2_^•−^) [17]. These short-lived chemical species then react with surrounding cellular components and biomolecules, resulting in the alteration of cellular signaling pathways that will induce cell death [18].

As previously mentioned, tumor control by EBRT with X-rays has often proved inadequate, thus justifying the development of therapeutic agents that can effectively improve the therapeutic index of RT, known as radiosensitizers. One strategy is to increase the effects of RT by acting on one of the 6 Rs of radiobiology. For example, inhibitors of the poly-ADP ribose polymerase (PARP) enzyme were found to prevent the repair of DNA damage and to be vasoactive (repair and reoxygenation) [19,20]. EBRT can also be used in combination with chemotherapeutics such as 5-fluorouracil, which prevents cells from entering the S-phase (redistribution) [21,22], or platinum compounds that interfere with DNA repair mechanism (repair) [23,24]. However, these radiosensitizers do not have a selective action in tumor cells, and their systemic biodistribution is associated with normal tissue toxicity, thus limiting their potential benefits [23,25,26].

Among radiosensitizers, agents composed of high-Z elements can increase the X-ray dose delivered because of their greater mass energy-absorption coefficients; they are known as radioenhancers. If sufficiently accumulated in tumors, they can improve the differential in dose deposition between normal and tumor cells, and thus the therapeutic index of RT [27]. Following this physical interaction, agents composed of high-Z elements can induce a cascade of biological events that depend on their biodistribution and may result in cell death. Indeed, they have both a radioenhancement potential through a physical-enhancement mechanism, and a radiosensitizing potential by a biological modification of cells before ionizing radiation [2,28,29].

The first discovery of radiation dose enhancement with high-Z elements dates back to the 1980s, where iodine contrast agent was found to sensitize cells to X-rays [2]. Lately, nanotechnologies provided new tools in this field, with the development of metal-based nanoparticles (NPs) as nano-radioenhancers or nano-radiosensitizers. The first preclinical proof-of-concept of nano-radiosensitization was shown with gold NPs by Hainfeld et al. in 2004 [30]. However, since this pioneering work and two decades of intensive research in this field, products based on gold NPs are still transitioning into clinical trials, and have never been clinically evaluated in combination with RT [31]. Several other metal-based NPs have been studied in cancer research so far, with the metal being lanthanum (Z = 57) [32,33], gadolinium (Z = 64) [34,35], hafnium (Z = 72) [36], platinum (Z = 78) [37], or bismuth (Z = 83) [38]. Nano-radioenhancers have broad therapeutic potential, as they can increase the dose delivered to tumors originating from a wide variety of organs. Indeed, their mechanism of action is not related to a specific biological target but based on radiation–matter interactions and influenced by their biodistribution. Moreover, in vitro studies demonstrated their ability to increase oxidative stress, DNA damage, and tumor cell death, while in vivo experiments showed their capacity of inducing tumor regression and improving animal survival [1,2].

Nowadays, two metal-based nano-radioenhancers are evaluated in Phase 2/3 clinical trials: NBTXR3 and AGuIX [39]. NBTXR3 is a 50 nm diameter NP made of hafnium oxide that is developed by the Nanobiotix company and designed for intratumoral injection. Its safety was confirmed in a Phase 1 clinical trial [40], and the first elements of therapeutic efficacy were obtained in a Phase 2/3 clinical trial [41], with both studies being conducted for the treatment of locally advanced soft-tissue sarcoma. NBTXR3 is currently being evaluated in a pivotal Phase 3 trial for the treatment of head and neck cancer (NCT04892173).

**Figure 1:**
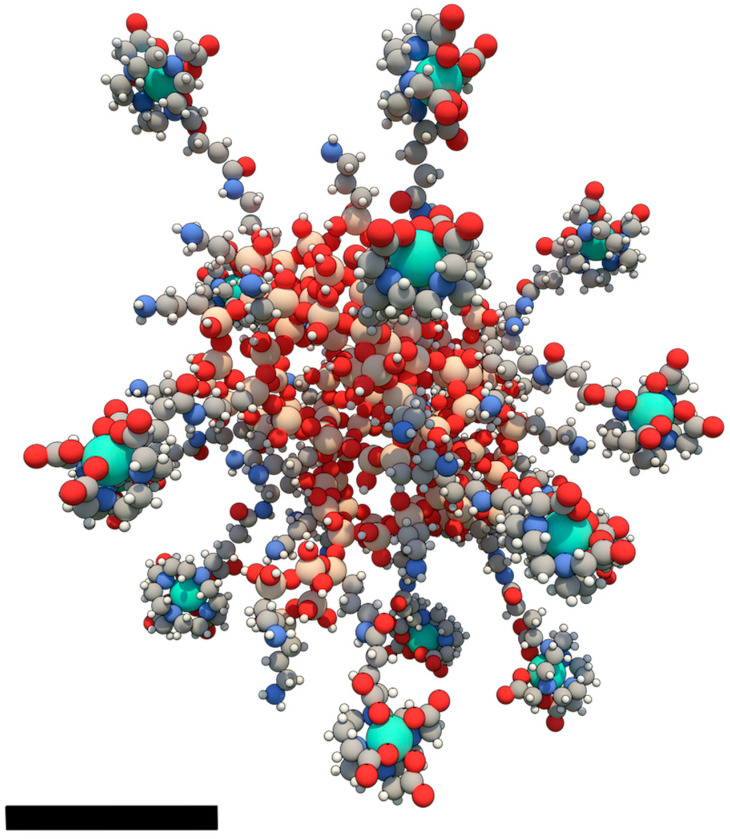
Schematic AGuIX structure. Three-dimensional representation of AGuIX modeled with the Avogadro software (v. 1.2.0). Polysiloxane core (Si, light orange; O, red; H, white; C, grey; N, blue) surrounded by covalently grafted chelates of gadolinium (Gd, cyan). Scale bar = 1 nm. Reprinted from Rocchi, 2022 [43].

Activation and Guidance of Irradiation by X-ray (AGuIX) is an ultrasmall gadolinium-based NP of 5 nm in diameter, developed by the NH TherAguix company and formulated for intravenous injection. Its structure consists of a polysiloxane matrix grafted with cyclic gadolinium chelates (Figure 1). AGuIX is prepared according to an original top-down process consisting in the formation of an intermediate core–shell nanostructure. This step is followed by core dissolution in acidic aqueous medium, and the process ends with a purification by tangential flow ultrafiltration [42]. To date, AGuIX production has been successfully transferred to industrial plants for good manufacturing practice (GMP) production, with some adaptations.

AGuIX is considered as a theranostic agent with interesting properties for both imaging and RT. First, AGuIX takes advantage of the well-described enhanced permeability and retention (EPR) effect [44] to efficiently reach the tumor after systemic injection [3]. Then, the presence of gadolinium (Z = 64) provides magnetic resonance imaging (MRI) positive contrast, as well as radioenhancing properties. Indeed, AGuIX was able to improve the efficacy of RT in vitro and in vivo on a wide range of cancer types, including glioma, brain metastases, pancreatic cancer, and many others [35]. Taken together, these results underline the pan-cancer therapeutic potential of AGuIX, which has the capacity of treating all tumors receiving RT, whatever the organ of origin.

Regarding its administration in human patients, AGuIX has been successfully evaluated in two Phase 1 clinical trials, respectively targeting brain metastases (NANO-RAD, NCT02820454) [45,46] and advanced cervical cancer (NANOCOL, NCT03308604) [47]. The NANO-RAD trial demonstrated precise and specific biodistribution in brain metastases, renal elimination, and clinical benefit for 13 of the 14 treated patients [45,46]. The NANOCOL trial revealed the tolerance of multiple AGuIX administrations in combination with cisplatin-based chemotherapy and brachytherapy and yielded excellent local control, with all patients achieving complete regression of the primary tumor [47]. In both cases, the relationship between AGuIX concentration and tissue changes was evaluated as a first hint of radiosensitization. AGuIX is currently evaluated in several Phase 2 and 1b/2 clinical trials, including two for the treatment of brain metastases (NANORAD 2, NCT03818386; NANOBRAINMETS, NCT04899908), one for glioblastoma (Nano-GBM, NCT04881032), and one for pancreatic cancers and lung tumor lesions (Nano-SMART, NCT04789486). To date, more than 220 patients have been injected with AGuIX, with a good profile of local and systemic tolerance.

The results obtained so far suggest that AGuIX could be a game-changer for RT-treated cancer patients. Indeed, this nanomedicine improves the therapeutic index of RT while being well tolerated after systemic injection in patients.

The goal of this review is to provide the best up-to-date understanding of the mechanisms of action of AGuIX in combination with radiation therapy, in particular, conventional EBRT.

First, as AGuIX localization and biodistribution properties are thought to play a pivotal role in these complex mechanisms, the first part will be devoted to them. Then, the complex mechanisms of action of AGuIX will be described, starting from physical interaction with incoming radiation, followed by an intermediate chemical stage of ROS production, and ultimately triggering a cascade of biological responses that result in tumor cell death.

## 2. Biodistribution: A Key Factor Influencing the Mechanisms of Action

### 2.1. Tumor Penetration

**Figure 2:**
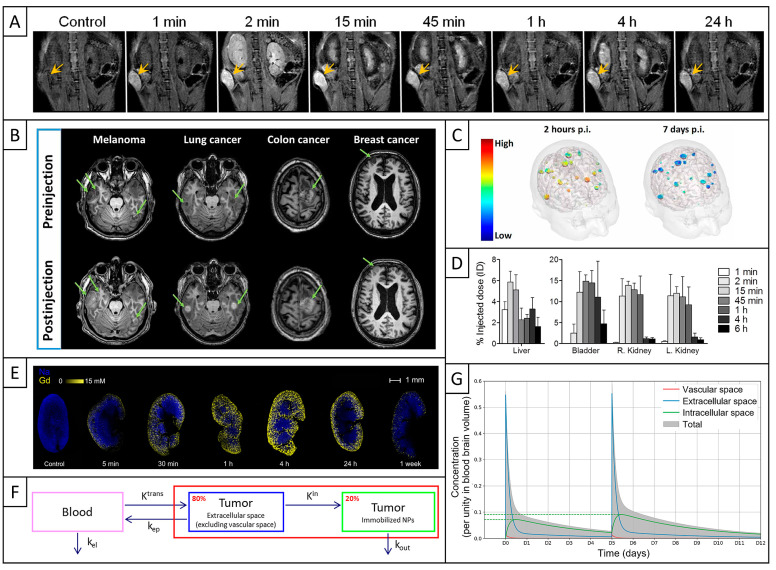
Biodistribution and elimination after intravenous injection. (**A**) T_1_-weighted magnetic resonance imaging (MRI) at various time points after AGuIX intravenous injection in Capan-1 tumor-bearing mice. The orange arrows are pointing the pancreatic tumor. Adapted from Detappe et al., 2016 [52]. Copyright © 2016, with permission from Elsevier. (**B**) Three-dimensional T_1_-weighted MRI obtained before (first row) and 2 h after (second row) AGuIX intravenous injection in human patients with multiple brain metastases from four types of primary tumors. The green arrows are pointing highlighted metastases. Adapted from Verry et al., 2020 [45]. (**C**) Color-encoded MRI signal enhancement (SE) in brain metastases, 2 h (**left**) and 7 days (**right**) after AGuIX intravenous injection in a patient with multiple brain metastases from lung cancer. Three-dimensional visualization of patient’s brain was performed using the BrainVISA/Anatomist software (v. 4.6.1) developed at NeuroSpin (CEA, Saclay, France). Reprinted from Verry et al., 2020 [45]. (**D**) Quantification of MR signal in healthy organs of Capan-1 tumor-bearing mice at various time points after AGuIX intravenous injection, represented as a percentage of the injected dose (% ID). A region of interest was drawn across the vital organs, and the T_1_ contrast was measured and correlated to its respective calibrations (ParaVision (v. 5.1)). All data are represented as mean ± standard deviation (SD), *n* = 3 animals per group. Adapted from Detappe et al., 2016 [52]. Copyright © 2016, with permission from Elsevier. (**E**) Laser-induced breakdown spectroscopy (LIBS) quantitative imaging of gadolinium (Gd, yellow) and sodium (Na, blue) elements in the kidney of a non-tumor-bearing mouse at different time points after AGuIX intravenous injection. For elemental quantification, standards containing AGuIX were embedded in the same EPON resin as for kidneys, at five concentrations ranging from 1 to 40 mM. Spatial resolution = 40 µm. Scale bar = 1 mm. Adapted from Sancey et al., 2015 [57]. (**F**) Schematic representation of the multi-compartment model proposed to model the kinetics of AGuIX distribution in tumor tissue, adapted from the three-compartment model of Tofts. The model includes a vascular compartment (pink) and an extravascular compartment (red) that comprises an intracellular compartment (blue) and an extracellular one (green). All constants are expressed in min^−1^; transfer constants are *K^trans^* and *K^in^*; and rate constants are *k_el_*, *k_ep_*, and *k_out_*. Self-produced figure. (**G**) Simulation from the proposed multi-compartment model representing the evolution of AGuIX concentration in the three major compartments over time and after two intravenous injections at day 0 and day 5. The simulation was fitted on experimental data from a rat glioma model. Dashed lines represent the maximum amount of AGuIX accumulated in the intracellular compartment after each injection. Self-produced figure.

Before delving into the details of the physical, chemical, and biological mechanisms resulting from the interaction between AGuIX and ionizing radiation, it is essential to take a broader look at the fate of AGuIX after its intravenous administration. AGuIX effectively accumulates in the tumor via the EPR effect due to increased tumor permeability, as demonstrated in many preclinical in vivo studies (Figure 2A, [48,49,50,51,52,53,54,55]). Regarding the kinetics, a positive contrast enhancement was observed on MRI in tumors from the first minute after AGuIX intravenous injection [52,56].

The ability of AGuIX to accumulate in tumors was confirmed in several clinical trials on various indications, including brain metastases (Figure 2B, [45,46,58]), glioblastoma [59], and cervical cancer [47]. A first clinical trial (NANO-RAD, Phase 1) evaluated AGuIX biodistribution in 15 patients with multiple brain metastases. In this dose-escalation study, AGuIX was administered by a single intravenous injection at doses ranging between 15 and 100 mg/kg, 4 h before the first whole-brain radiation therapy (WBRT) session. Its magnetic properties differ from clinical Gd-based contrast agents like Dotarem^®^ (Guerbet, Villepinte, France) due to its larger diameter and molecular weight, resulting in a higher longitudinal relaxivity (r_1_) of 8.9 mM^−1^·s^−1^ compared to 3.5 mM^−1^·s^−1^ for Dotarem^®^, each measured per Gd^3+^ atom and at 3 T [45]. MRI results obtained 2 h after AGuIX injection demonstrated that AGuIX was associated with a noticeable signal enhancement (SE) for all measurable metastases. Indeed, the mean MRI SE demonstrated a linear correlation with the dose of AGuIX injected, with higher doses associated with stronger signal in the metastases. For the patients receiving the highest dose (100 mg/kg, *n* = 3), the mean MRI SE reached 120.5%, and the AGuIX concentration varied between 8 and 63 µM of Gd^3+^ ions (*n* = 24 metastases) [45]. These results were further confirmed by the first outcomes of another clinical trial (Nano-GBM, Phase 1b/2) that evaluated AGuIX in eight patients with glioblastoma. An MRI SE was observed in the tumor of all patients receiving the highest dose (100 mg/kg, *n* = 6) after AGuIX injection, with higher Gd concentrations at the border of the tumor mass than in the center highlighting the morpho-functional heterogeneity of such tumors. The mean concentration of Gd^3+^ varied between 54.5 and 160.3 μM in the gross tumor volume [59]. Last, in the Phase 1 clinical trial, a correlation was established between AGuIX intratumoral uptake and the reduction of metastasis volume, clinically highlighting the role of AGuIX in radiosensitization [46].

### 2.2. Retention Properties

After entering the tumor, AGuIX gradually accumulates before being eliminated over time [49,51,52]. Regarding the retention, the gadolinium element, representative of the presence of AGuIX, was still detected in rat gliomas 24 h after AGuIX injection, by both MRI and X-ray fluorescence methods [56]. A signal persistence at 24 h was also observed in other tumor types, including pancreatic, lung, and breast cancer, confirming the prolonged retention of AGuIX in the tumor [35]. These specific retention properties notably differ from those of clinically approved Gd-based contrast agents like Dotarem^®^. In a preclinical study, both agents increased contrast enhancement in rat gliomas in the first minutes after administration. However, AGuIX reached a plateau 7 min post-injection and was still observed 24 h later, whereas Dotarem^®^ exhibited a peak signal 1 min after injection and decreased steadily over the subsequent minutes [60].

The specific retention properties of AGuIX were confirmed clinically, with a persistence of MRI SE in brain metastases 7 days after AGuIX injection, highlighting a delayed clearance of the nanomedicine from the tumors (Figure 2C, [45]). The mean plasma half-life was very similar between studies for the 100 mg/kg dose, with T_1/2_ = 1.21 h in NANO-RAD [46] and T_1/2_ = 1.41 h in Nano-GBM [59]. Last, AGuIX was progressively eliminated, as there was no longer noticeable MRI SE 4 weeks after injection [46].

Taken together, these results emphasized the need to consider a repeated weekly administration of AGuIX in following clinical trials to better fit fractionated radiation regimens [46]. Thus, subsequent clinical trials were designed with multiple AGuIX injections: 2 in Nano-SMART and NANOBRAINMETS, 3 in NANORAD 2 and NANOCOL, and 4 in Nano-GBM. In addition, a patent has been filed to cover the specific schedule of administration of AGuIX combined with RT [61].

### 2.3. Elimination from the Body and Safety

As an ultrasmall NP, AGuIX behaves in a so-called “quasi-molecular” regime, allowing a fast circulation, an evasion from the first-line immune system, and a rapid excretion by the kidneys [62,63]. Indeed, after its accumulation in tumors, AGuIX is rapidly eliminated through the kidneys, with a gradual signal decrease after several hours reflecting efficient elimination from the body via urine (Figure 2D,E, [48,49,50,52,53,57]). AGuIX was also found in smaller proportions in the liver, but its level decreased over time, indicating no specific hepatic accumulation (Figure 2D, [48,49,52,53]). Clinical results confirmed a rapid renal clearance with a mean urinary excretion of 54% during the first 24 h after AGuIX injection, with the remaining part being eliminated the week after [46].

Regarding the safety of the nanomedicine, preclinical in vivo studies showed that there was no extravasation in the healthy brain [35]. Regulatory safety pharmacology studies were performed in rats and non-human primates (Cynomolgus monkeys). These studies emphasized the importance of AGuIX administration protocol, which includes pH adjustment at 7.2 ± 0.2 and formulation 1 h before injection. In both cases, no major adverse effects were observed up to the higher dose tested of 450 mg/kg in rats and 750 mg/kg in non-human primates [64]. Indeed, AGuIX did not affect neurologic function or cardiac and respiratory rates, and no hypersensitivity reactions were reported.

Clinically, no significant variation in MRI signal was detected in healthy brain tissue after AGuIX administration [46,59]. Moreover, clinical data were consistent with regulatory studies, with no acute grade 3 or 4 adverse effects attributed to AGuIX [45,47,59]. Last, it is worth mentioning that the potential adverse effects related to AGuIX are only expected after RT and correspond to an increase in RT-induced damage in the irradiated region.

### 2.4. Modelling the Kinetics of Accumulation and Retention

The long retention properties of AGuIX shown in the NANO-RAD trial yield an estimated half-life of 2 to 3 days in tumors when assuming a classical exponential decay model [45]. However, short-term imaging performed in the NANORAD 2 trial indicates a clearance of the product within a few hours (data in publication under preparation). These data suggest that while a large proportion of NPs are rapidly cleared after injection, a small fraction remains immobilized for a long time. The discrepancy between datasets when using a simple one-compartment model highlights the need for more complex models.

Thus, to model the kinetics of AGuIX distribution in tumor tissue, a novel multi-compartment model was proposed. This model extends and adapts the classical three-compartment model of Tofts [65], and it has already been validated through preliminary experiments in a rat glioma model. The proposed model includes three compartments: the vascular compartment, the tumor cell compartment (~20%), and the extracellular extravascular space (EES, ~80%), with the last two being part of the extravascular compartment (Figure 2F). Constants have been defined according to standard notations of compartment modeling in pharmacokinetics, with transfer constants being *K^trans^* and *K^in^*, and rate constants being *k_el_*, *k_ep_*, and *k_out_*, all of them expressed in min^−1^.

Since AGuIX is administered via intravenous injection, it is initially only localized in the bloodstream, and then it rapidly extravasates across the blood–brain barrier and accumulates in the extracellular matrix (*K^trans^*). Meanwhile, AGuIX is progressively eliminated from the blood (*k_el_*), with a plasma half-life of 1 to 2 h [46,59]. Moreover, a proportion of AGuIX transfers from the EES to the blood circulation (*k_ep_*). Contrary to the Tofts model, which assumes that gadolinium contrast agents do not enter cells, it is hypothesized here that AGuIX is internalized into cells, based on preclinical results that will be discussed later (Section 2.5, [48,50,51,55,66,67,68,69,70,71,72]). Thus, AGuIX progressively transfers from the extracellular to the intracellular compartment, leading to long-term immobilization in the latter (*K^in^*). Last, it was assumed that NPs are not immobilized indefinitely and may be progressively eliminated from the intracellular space, potentially due to cell death mechanisms (*k_out_*).

The next step consisted in solving the model’s equations and adjusting the transfer rates to match experimental data from tumor-bearing animals. The kinetics of AGuIX distribution across individual compartments was highly consistent with clinical findings, with both rapid plasma clearance and NP retention for 7 days. This model now enables simulations of multi-injection protocols, assuming that multiple injections lead to an accumulation of AGuIX (Figure 2G). These simulations align with observations from the NANORAD 2 clinical trial and allow for precise estimations of AGuIX concentration in each compartment.

To sum up, AGuIX accumulates in tumors for several hours after its intravenous injection, before being cleared via the renal route. Its specific retention properties can be modelized by a multi-compartment model that explains both its rapid elimination from the bloodstream in the first hours and the long persistence in the tumor for several days. This specific fate is highly dependent on time and space, two parameters that must be carefully considered when defining the delay between AGuIX injection and RT. Therefore, particular attention has been paid to the capacity of AGuIX to be internalized in tumor cells and the associated kinetics, as well as its localization in several subcellular compartments.

### 2.5. Cell Internalization and Subcellular Localization

The cellular uptake of high-Z NPs is driven by their physicochemical properties, including size, shape, surface charge, and biochemical properties. The interactions between these nano-radiosensitizers and cancer cells, as well as their subcellular localization, are key parameters influencing treatment efficacy [73].

When physical simulations were performed to assess the effects of AGuIX based on its measured intracellular concentration, the calculated radiosensitizing effect was not sufficient to explain the cytotoxicity observed experimentally. This discrepancy might be clarified by a deeper understanding of AGuIX distribution at the cellular level, as its subcellular localization may be a key factor influencing its radiosensitizing potential.

**Figure 3:**
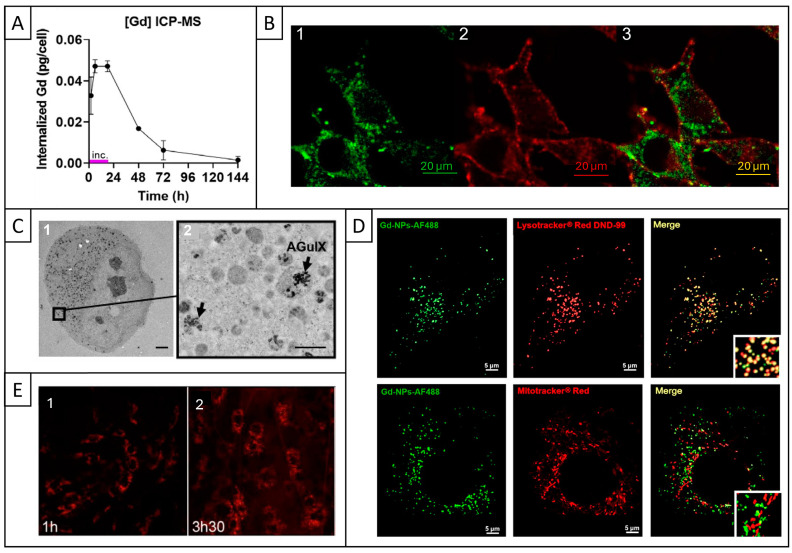
Intracellular uptake and localization. (**A**) Cellular uptake of AGuIX in SK-OV-3-luc cells represented as the quantity of gadolinium as a function of time and quantified by inductively coupled plasma–mass spectrometry (ICP-MS), including an 18-h incubation period (inc., represented by the purple line). All data are represented as mean ± SD and are a result of 3 independent experiments. Reprinted from Diaz Garcia-Prada et al., 2023 [50]. (**B**) Fluorescence images representing the intracellular localization of AGuIX–fluorescein isothiocyanate (FITC) in B16F10 cells, obtained by confocal microscopy. AGuIX-FITC labeled in green (**1**), plasma membranes in red (**2**), and merged image in (**3**). Scale bar = 20 µm. Adapted from Kotb et al., 2016 [51]. (**C**) Transmission electron microscopy (TEM) images showing the internalization of AGuIX (black arrow) in endosomal vesicles located in the cytoplasm of PANC-1 cells. Scale bar = 5 μm [3000×, (**1**)]. Scale bar = 1 µm [25,000×, (**2**)]. Adapted from Detappe et al., 2015 [66]. (**D**) Fluorescence images representing AGuIX-Alexa Fluor 488 (AF488, ThermoFisher) subcellular localization in SK-OV-3-luc cells in green (left), along with lysosomes (LysoTracker red DND-99 (L7528, Thermofisher), top middle) and mitochondria (MitoTracker red CMXRos (M7513, Thermofisher), bottom middle) in red. Merge images in right panels show the eventual colocalization in yellow. Scale bar = 5 µm. Reprinted from Diaz Garcia-Prada et al., 2023 [50]. (**E**) Fluorescence images from intravital two-photon microscopy representing AGuIX-FITC localization in tumor tissue of subcutaneous B16F10 tumor-bearing mice, 1 h (**1**) and 3.5 h (**2**) after intravenous injection. Adapted from Kotb et al., 2016 [51].

Several in vitro studies demonstrated that AGuIX was effectively internalized in tumor cells [48,50,51,55,66,67,68,69,70,71,72]. The intracellular uptake was quantified by assessing gadolinium content inside cells with spectrometry methods (Table 1 and Figure 3A).

Regarding its uptake mechanisms, AGuIX is internalized by endocytic mechanisms [69,73,74]. More specifically, one study observed the presence of membrane invagination along with clusters of AGuIX in endosomes and lysosomes, indicating clathrin-mediated endocytosis [74]. Another study explored the internalization of Gd-based NPs similar to AGuIX and showed their agglomeration near the cell, as well as lamellipodia extensions of the plasma membrane toward the extracellular medium, representative of micropinocytosis [75]. Regarding internalization kinetics, AGuIX is already present inside cells as early as 30 min [48,66] or 1 h [67] after incubation, and still in significant proportions 48 h later [50,66]. One study demonstrated that AGuIX uptake increased with its concentration, with values that were 4 and 7 times higher at 1.5 mM and 2 mM compared to 0.8 mM [69]. Among the various studies evaluating AGuIX intracellular uptake, results showed a concentration ranging from 0.04 to 0.22 pg of Gd/cell [50,66,68,71,72].

Although the internalization of AGuIX largely influences its radiosensitizing capacities, many other parameters may also be involved. For example, a study showed that cells incubated with AGuIX presented higher survival after irradiation when their medium was changed just before irradiation, highlighting the contribution of NPs that are not internalized into the cells [66]. Moreover, another study showed that the level of radiosensitization did not increase with NP uptake [71]. Indeed, some long-range effects beyond the primary physical interactions may impact AGuIX radiosensitization, including chemical and biological processes.

At the same time, numerous studies also investigated the subcellular localization of AGuIX through microscopic observations (Table 2).

In most of the studies, AGuIX was observed in the cell cytoplasm (Figure 3B), either free in the cytosol [68], in the form of clusters (Figure 3C, [51,55,68,69,70]), or inside endosomal vesicles [51,66]. Regarding subcellular structures, AGuIX was not observed in the nucleus [50,67,68,70] or in the mitochondria (Figure 3D, [50,68,69]). Last, AGuIX demonstrated a high colocalization with lysosomes (Figure 3D, [50,68,69]), where its uptake increased from 2 to 6 h and decreased after 18 h [50]. These vesicles are essential intracellular organelles that play a key role in the degradation of biomolecules [73].

In addition, one in vivo study showed that AGuIX was mainly located in the extracellular matrix in the first hour post-injection, and then appeared as small dots within the tumor cells corresponding to an internalization into vesicles, consistent with in vitro studies (Figure 3E, [51]). Thus, AGuIX is efficiently internalized inside tumor cells, and more specifically located in lysosomes.

### 2.6. Cytotoxicity

Regarding the biological toxicity of the nanomedicine itself, both cell and animal studies demonstrated that without RT, AGuIX did not alter any biological function after its administration. Indeed, numerous in vitro studies showed that AGuIX did not modify ROS generation; DNA damage; cell cycle repartition; or any cell death mechanisms, including apoptosis, autophagic cell death, and ferroptosis [48,51,52,53,68,71,76,77]. One study showed a slight increase in the number of γ-H2AX foci per B16 cell from ~9 to 15 in the presence of AGuIX compared to control cells, but no influence on ROS level, cell apoptosis, or immunogenic cell death [78]. Moreover, in vivo studies demonstrated that AGuIX did not change animal body weight, clinical symptoms, tissue microanatomy, or DNA damage [48,52,53,77].

In brief, AGuIX is effectively internalized in various types of tumor cells and located in the cytoplasm, including subcellular compartments, such as lysosomes. Since the NP has never been observed in the nucleus, its radiosensitizing effect is more likely caused by indirect DNA damage. In addition to its internalization capabilities, the radiosensitization of AGuIX is influenced by a large number of RT-induced mechanisms, including physical interaction, oxidative stress, DNA damage, cell death process, and immune response, which are described thereafter.

## 3. Initial Interaction: Any Physical Increase in the Radiation Dose?

### 3.1. Interaction of Ionizing Radiation with Matter

**Figure 4:**
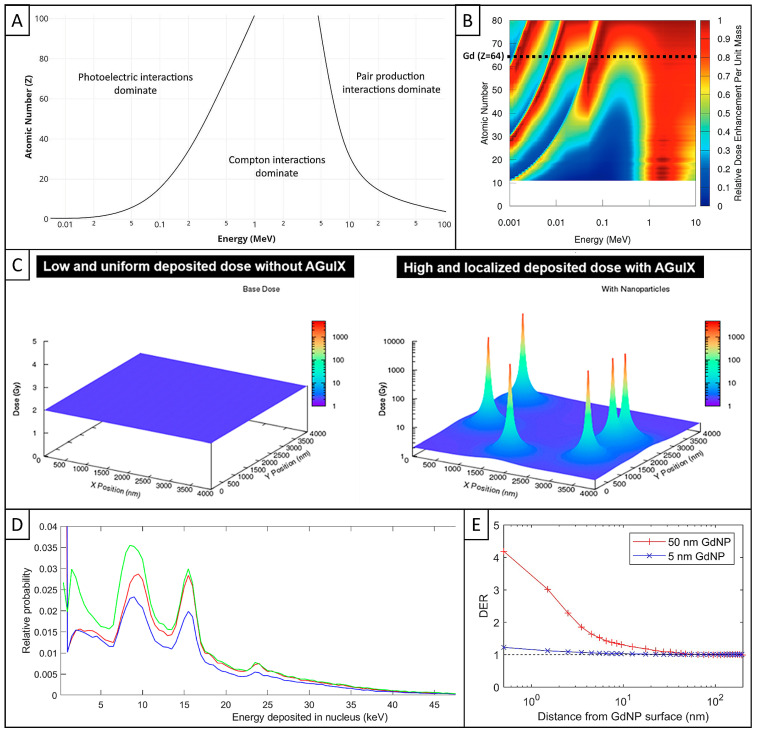
Physical interaction with ionizing radiation. (**A**) Three main photon interactions with matter as a function of photon energy and atomic number. Reprinted from Mott and Daniel, 2021 [81]. Copyright © 2021, with permission from Elsevier. (**B**) Relative dose enhancement per unit mass as a function of photon energy and atomic number. Adapted from McMahon, Paganetti, and Prise, 2016 [82], with gadolinium as dashed line (Z = 64). (**C**) Simulation of the local dose deposition after conventional external beam radiation therapy (EBRT) at 2 Gy and 80 keV, without (**left**) or with AGuIX (**right**), according to the work of McMahon et al., 2011 [27] Adapted from Lux et al., 2015 [83]. Copyright © 2015, with permission from Elsevier. (**D**) Distribution of energy deposited in the nucleus from one photon-ionizing event for AGuIX irradiated with 250 kVp beam. Nanoparticle (NP) distribution is uniform around the nuclear membrane, in green; uniform throughout the entire cell, in red; and uniform in the cytoplasm only, in blue. Adapted from Yan et al., 2021 [80]. (**E**) Radial dose enhancement ratio (DER) around AGuIX irradiated as a single particle (5 nm) or as a cluster (50 nm). Dashed line represents the absence of dose enhancement. Reprinted with permission from Wu et al., 2023 [84].

Interaction of ionizing X-ray photons with matter is relatively well described in the literature, elements constituting matter being defined by their absorption cross-section, contributing to the attenuation of incident photons through absorption and scattering [79,80,81]. As a function of the atomic number (Z) of the element and the energy (E) of the photons, various physical interactions may occur with a defined probability. The three main interactions are the photoelectric effect, the Compton effect, and the pair production (Figure 4A [81]). The photoelectric effect is characterized by the emission of an inner-shell photoelectron and the subsequent emission of Auger electrons, called Auger cascade. For a given E, its probability of occurrence depends on Z^3^. The Compton effect is characterized by the scattering of an incident photon associated with the emission of an outer-shell electron, and does not depend on Z. The pair production is associated with the production of a pair of particles, an electron and a positron, and it depends on Z. Secondary particles, including electrons, as well as eventual scattered photons resulting of such primary interaction, may be further involved in ionization events of the same nature [81]. Based on their respective energy, the contribution of the different particles to dose deposition will differ, with photoelectrons and Compton electrons (up to few keV energy range) depositing their energy in the micrometer range, while Auger electrons (few tens of eV energy range) do so in the nanometer range [2]. Unless they are generated within the nucleus, these secondary species will mostly trigger DNA damage through indirect mechanisms. Finally, these processes of interaction with matter define the dose that will be deposited locally and are responsible for biochemical events that subsequently lead to cell death.

Regarding the correlation with photon energy, the photoelectric effect depends on 1/E^3^ for a given Z and therefore becomes predominant at lower energies. In addition, absorption will be maximum at energies just above K-shell binding energy, also called K-edge energy. For instance, K-edge energies for gold and gadolinium are 80.7 and 50.2 keV, respectively [85]. For their part, the Compton effect depends on 1/E, and the pair production becomes predominant at very high energies.

### 3.2. Key Factors of Radioenhancement of High-Z NPs Beyond Limits

The benefits of using high-Z elements for enhancing dose deposition have been demonstrated by relatively simple calculations based on National Institute of Standards and Technology (NIST)-given mass energy absorption ratios of elements [27]. These calculations are based on a macroscopic scale, i.e., considering a hypothetic homogeneous distribution in space of high-Z elements that does not reflect in vivo reality. The analysis is presented in Figure 4B, where bands corresponding to M-, L-, and K-shell absorption can be seen at incident low energies to show the theoretical impact of high Z elements on physical dose enhancement, highlighting gadolinium as the main element of AGuIX [80]. This physical dose enhancement is caused by the generation of secondary particles, including photoelectrons with relatively high energy, followed by a cascade of Auger electrons with lower energy [1,27].

NPs and in particular inorganic or hybrid NPs appear as efficient vectors to carry these high-Z elements in vivo, taking advantage of their engineerability and potential to target tumor cells. As a result of the macroscopic view, the mass concentration of NPs in the tumor can be seen as one of the key parameters driving radiation dose enhancement. Whereas theoretical models on gold NPs only predicted a significant dose enhancement above mass concentrations of 1%, experimental studies showed significant radiosensitization at concentrations that were orders of magnitude smaller [27]. In the particular case of AGuIX, a significant radiosensitization was demonstrated at gadolinium mass concentrations ranging from 0.001 to 1% [83]. Efficacy data were obtained both in vitro and in vivo, in, respectively, nine and six tumor models issued from various tumor cell lines, and they were then clinically confirmed in the NANO-RAD Phase 1 clinical trial [46]. Indeed, patients with brain metastases were treated with a combination of AGuIX injection and WBRT, and the study demonstrated a correlation between MRI-based gadolinium concentration measurements and tumor volume reduction. More recently, the AGuIX concentration was measured in the cervical tumors of four patients treated with a combination of AGuIX and cisplatin-based chemoradiation, using MRI T_1_ signal change (NANOCOL Phase 1 clinical trial), in agreement with mass spectrometry results on respective biopsies [86]. Three-dimensional cell culture models were then used to mimic clinical conditions, and the results supported AGuIX radioenhancing potential, confirming the gap with theoretical analysis of purely physical dose enhancement. Altogether, preclinical experimental data confirmed AGuIX suitability for radiosensitization following intravenous injection, although this method allows less than 1% of the injected dose to accumulate in the tumor. This statement was further confirmed by clinical data, demonstrating a promising local tumor control and a clinical benefit for patients receiving AGuIX with the SOCs [47]. Although these results need to be confirmed by the Phase 2 clinical trials currently underway, there are strong assumptions that concentration is a parameter influencing AGuIX radiosensitization but may not be a key factor.

Another important parameter that strongly influences radioenhancement is the composition of the NP itself, its structure, and its size. Simulations from McMahon et al. demonstrated that, at the same metal concentration, gold NPs induced higher local dose deposition as their size decreased, because of their larger surface area to volume ratio. Indeed, the majority of the local dose deposition results from events occurring within a thin shell at the surface of the NP [27].

Regarding AGuIX, this ultrasmall NP is composed of gadolinium ions that are chelated by 1,4,7,10-tetraazacyclododecane-1,4,7,10-tetraacetic acid (DOTA) derivatives covalently bound to a polysiloxane matrix. AGuIX carries, on average, 15 atoms of gadolinium, which enhances the probability of interaction with X-rays. In particular, this has been highlighted in simulations demonstrating higher local dose deposition for heterogeneous and clustered Gd-atom distribution (as in AGuIX) compared to isolated atoms [87]. In addition, gadolinium atoms are distributed at the surface of the NP in forms of chelates and directed toward the biological medium, thus allowing the escape of secondary particles involved in further cytotoxic ionization events [82].

For photon-beam energy, most of the simulations demonstrated that physical dose enhancement with high-Z elements could only occur at low-energy X-rays (keV) [88,89]. While theoretical enhancement factors are indeed lower for MeV than for keV photons, both in vitro and in vivo experiments demonstrated promising radiosensitization of high-Z NPs at clinical energy [1]. In clinics, EBRT is usually delivered with megaelectronvolt (MeV) photon beams, with an incident beam containing a broad spectrum of photons, with energy ranging from few tens of keV to a few MeV [80].

In vitro, the efficacy of high-Z NPs in combination with ionizing radiation is evaluated through clonogenic assays that allow the extraction of ratios and factors of radiosensitization from the linear quadratic (LQ) fitting of the cell survival curve, and in vivo, through animal survival and tumor control [90]. Following the LQ model, radiobiological parameters α and β are defined according to the following equation: SF(D) = e^−(αD+βD^2^)^, where SF is the survival fraction; D is the radiation dose; and α and β are, respectively, associated with direct and indirect radiation-induced damage [91]. Sensitizing enhancement ratio (SER) in another key radiobiological parameter used to quantify the radiosensitizing effect of NPs and defined as the ratio of the area under the survival curve of control cells (RT alone) to that of treated cells (RT and NPs). Values of SER above 1 indicate that the NPs enhance cell death in comparison to radiation alone, indicating some radiosensitizing effect [28].

For AGuIX, SER from clonogenic assays ranged from 1.1 to 2.5 at keV energies and from 1.1 to 1.7 at MeV energies [83]. Moreover, in clonogenic assays performed on F98 cells, AGuIX induced a strong radiosensitization at 1.25 MeV (γ-rays, ^60^Co source), whereas the theoretical Monte Carlo (MC) simulations predicted no effect at this energy [87]. Therefore, whatever the energy, the observed damage was always greater than that expected from theoretical calculations based on macroscopic physical dose enhancement [80].

Detappe and coworkers evaluated the impact of delivering photon beams with or without a flattening filter (flattening filter-free, FFF) [53]. A flattening filter can be used to induce a more uniform radiation dose distribution, but it also reduces the number of low-energy photons. Indeed, MC simulations demonstrated that the 6 MV-FFF beam contains 2.6 times more low-energy photons (<100 keV) than the 6 MV beam [53]. Interestingly, the study demonstrated that the 6 MV-FFF beam resulted in an improved outcome compared to the 6 MV beam. In greater details, when combined with AGuIX, the 6 MV-FFF beam increased cell survival, improved pancreatic tumor-bearing mice survival, and reduced tumor volume. However, it is worth mentioning that the experiments were carried out on subcutaneous tumors, which are favorable conditions for the 6 MV-FFF effect due to their superficial localization and low risk to surrounding healthy tissue. This study confirmed the added value of irradiation with keV photons, but also that valuable radiosensitization can be reached with a clinical irradiator.

### 3.3. AGuIX Simulations with Different Models

To better predict experimental data using NPs, computational models have been developed, correlating the primary physical interaction with cell SFs. Most of them were conducted using MC simulations and focused on gold NPs [27,28,79,89]. However, simulations often failed to predict experimental radiosensitization that finally exceeded theoretical predictions, and models have been increasingly improved to get closer to the biological reality.

Without NPs, irradiation of tumor cells with low-LET photons leads to sparsely ionizing events that can be represented by an homogeneous dose distribution in space, once simulated (Figure 4C (left), [83]). Based on this consideration, many studies have been performed at the macroscopic scale to simulate radiosensitization provided by high-Z NPs, which was ultimately not appropriate to model the strong local increase in dose deposition around the NPs. Indeed, when dose deposition calculations were carried out in the close vicinity of NPs rather than averaged over the whole space, theoretical results began to better match experimental ones. Over time, basic macroscopic approaches have been replaced by biophysical models that take better account of NP parameters, such as structure, concentration, biodistribution, and relative biological effectiveness (RBE) against nuclear DNA.

About AGuIX, a Geant-4-based MC simulation study was performed at the nanoscale to compute the interactions with the NPs after an irradiation at 80 keV [83]. This computational study includes the local effect model (LEM) for better translating the microscopic energy deposition patterns following photoelectric interaction and the contribution of the generated Auger electrons. Indeed, some mechanistic models like the LEM have been developed to better understand the radiobiological impact of high-Z NPs, considering cell and tissue response to X-ray irradiation for biological modelling using microdosimetry concepts. This study showed that the presence of several gadolinium atoms close to each other made the formation of an Auger electron cascade under irradiation highly probable. The generation of secondary particles increased the energy deposition in the vicinity of the NPs, resulting in several dose nanopeaks, as shown in Figure 4C (right).

Another study performed clonogenic assays comparing AGuIX (GdNP) and conventional gadolinium-based contrast agent (GdCA), in parallel with simulating how the intracellular gadolinium distribution could impact dose enhancement using the PENELOPE MC code [87]. The simulations predicted a massive and localized emission of low-energy electrons around the NPs. Regarding dose enhancement factors (DEFs), the experimental values were always higher than computed ones for GdNP, whereas GdCA did not induce dose enhancement. A possible explanation relies on the fact that GdCA is homogeneously distributed in the extracellular matrix and probably not taken up by the cells, while GdNP is partially taken up in the cytosol in the form of clusters. The authors hypothesized that computed localized DEFs in lysosomes may be enough to trigger cell death.

Another study used the Geant4-DNA toolkit to set up the Bomb model, based on the theory of dual radiation action (TDRA) [80]. This model evaluates the microdosimetric consequences of the Auger cascade and considers that an NP can kill a cell with a certain probability after being triggered by photoelectric interactions. The simulations showed that ionization of AGuIX provides a greater cell-killing potential than that of gold NPs at approximately the same photon energy (250 kVp and 220 kVp, respectively). This may be attributed to the lower self-absorption of secondary Auger electrons by AGuIX due to its smaller size. The study also emphasized the strong dependence of radiosensitization on the intracellular distribution of the NPs, especially the distance to the nucleus (Figure 4D). However, even if the proposed Bomb model may be a potential candidate for a general framework that describes AGuIX radiosensitization, it failed to fully fill the gap with experimental data.

Another model extended LEM to AGuIX using the TOPAS-nBio toolkit, and the simulation was based on a nanoscale dose distribution in the nucleus under keV photon irradiation [84]. The study demonstrated that most of the dose enhancement was due to the contribution of Auger electrons. Moreover, it showed that clusters of NPs displayed a larger extent of dose enhancement than single NPs (Figure 4E), although clustering significantly reduced radial dose per interacting photon. Despite achieving reasonable agreement with experimental results, authors highlighted that deviation from the chosen simulation parameters, especially proximity of the biologically sensitive volume, would cause major disparity.

Last, an MC simulation performed with the TOPAS-nBio toolkit aimed to study the interaction of AGuIX with the radionuclide ^177^Lu in lysosomes, i.e., interaction with beta electrons of 487 keV, 384 keV, and 176 keV [50]. The results demonstrated that AGuIX did not significantly modify the absorbed dose in the modelled lysosomal volume. Moreover, the study showed a very low contribution of photoelectrons, whereas Auger electron production was increased by 13% in the presence of AGuIX, probably responsible for their observed radiosensitizing effect.

### 3.4. Conclusions

All the above-mentioned studies agreed on a strong local increase in energy deposition around AGuIX, mainly through the production of Auger electrons. Although these results represent a major step forward in understanding the dose deposition of AGuIX and, more generally, of NPs, they do not consider other parameters, such as secondary interactions and variations in the local concentration of AGuIX in the tumor as a function of biodistribution and time scale. Consequently, the experimental radiosensitization usually exceeded the theoretical one. Deciphering the mechanism of action of NPs requires more complex nanoscale and local models that are able to integrate experimental results in order that predictions come closer to in vivo situation.

To a large extent, radiosensitization of high-Z metal-containing NPs like AGuIX seems to not rely on a macroscopic physical dose enhancement but rather on localized dose enhancements attributed to Auger electrons. This physical interaction is able to trigger various biological mechanisms, leading to oxidative stress and ROS production, DNA damage, cell death, and immune activation, which will be described in the following sections.

## 4. Chemical Stage: Any Enhancement of ROS Production Under RT?

### 4.1. ROS Measurement in Biological Studies

As mentioned in the introduction, the therapeutic effect of RT mostly relies on an indirect mechanism, with the formation of ROS through water radiolysis. ROS are metabolic byproducts naturally produced by mitochondria and endoplasmic reticulum. Their high reactivity toward biological components acts as a signal to modulate the biological functions of the cells. Indeed, any imbalance in the antioxidant mechanism which regulates their level can induce oxidative stress that will damage cell components and eventually lead to cell death [92].

The most common ROS include hydroxyl radical (•OH), superoxide anion radical (•O_2_^−^), and hydrogen peroxide (H_2_O_2_). ROS measurement in real time is very challenging because of the very short half-life of these species, ranging from nano- to milliseconds. Indeed, secondary markers, such as radical scavengers, and especially fluorescent dyes, are more commonly used. After being added to the medium, these compounds react in situ with ROS and produce a detectable substance with a much longer lifespan. For instance, 2′,7′-dichlorofluorescein diacetate (DCFDA) is the most commonly used dye, as it is cell-permeable and sensitive to many different ROS and even nitric oxide (•NO) [93].

### 4.2. ROS Production Under RT by High-Z Metal-Based NPs

High-Z NPs may contribute to the enhancement of ROS production under irradiation through the local increase in the deposited dose and the associated production of secondary electrons. These secondary particles, mainly Auger electrons, interact with nearby oxygen-containing molecules—mainly water ones—and increase ROS production.

The level and nature of the ROS generated vary according to the subcellular, cellular, and tissular localization of NPs. As an example, ROS generation implied in ferroptosis has been shown to depend on lysosomal activity, which could be influenced by clusters of NPs [94]. ROS generation also depends on NP parameters, including size, shape, and surface chemistry [93], as well as injection route. Regarding NP size and shape, many studies emphasized the importance of surface area, higher in smaller NPs, as it maximizes NP interactions with X-rays and thus ROS generation. Moreover, increasing NP size is associated with greater self-absorption of secondary electrons generated under RT, reducing dose deposition and ROS generation. Last, structure and surface functionalization affect the stability, biocompatibility, cell uptake, and clearance of the NPs. In some cases, surface coating was shown to improve the transport of oxygen-based molecules to increase ROS generation [93]. Thus, the localization and intrinsic structural parameters of NPs have a strong influence on radiosensitization and ROS generation and may impact their mechanism of action.

### 4.3. ROS Production Under RT in the Presence of AGuIX

Several studies investigated the influence of AGuIX on ROS production, as summarized in Table 3 Regarding ROS measurement method, all the studies used fluorescent dyes, and the associated fluorescence signal was either analyzed by a plate reader or by flow cytometry.

All the studies reported that AGuIX further increased the fluorescence signal associated with various ROS probes under RT. It is worth noting that cancer cells exhibit high basal ROS levels as a result of an imbalance between oxidants and antioxidants, even without treatment [95].

**Figure 5:**
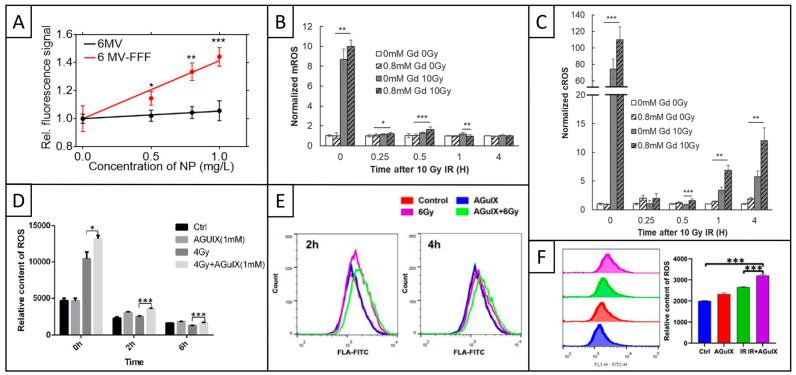
Reactive oxygen species generation. (**A**) Reactive oxygen species (ROS) measurement in Capan-1 cells quantified as the ratio of fluorescence after 4 Gy irradiation with and without different concentrations of AGuIX. All data are represented as mean ± SD. Statistical tests were performed using Kruskal–Wallis test. Reprinted from Detappe et al., 2016 [53]. (**B**,**C**) Kinetic study of mitochondrial ROS (mROS, (**B**)) and cytosolic ROS (cROS, (**C**)) generation in SQ20B cells under 10 Gy irradiation and after AGuIX incubation. Results were obtained by flow cytometry and normalized as a function of the non-treated and non-irradiated cells, and glutathione (GSH) was previously depleted. All data are represented as mean ± SD, with 2 independent experiments. Statistical tests were performed using Kruskal–Wallis test and Dunn’s multiple comparison test as post hoc. Used with permission of American Scientific Publishers, reprinted from Simonet et al., 2020 [68]; permission conveyed through Copyright Clearance Center, Inc. (**D**) Total fluorescence intensity, representing ROS levels in H1299 cells under 4 Gy γ-ray irradiation and after AGuIX incubation. All data are represented as mean ± SD, with 3 independent experiments. Reprinted with permission from Du et al., 2020 [48]. Copyright © 2020, American Chemical Society. (**E**) Total fluorescence intensity measured by flow cytometry and representing ROS levels in B16 cells induced under 6 Gy irradiation and after AGuIX incubation. Used with permission of Royal Society of Chemistry, reprinted from Song et al., 2022 [78]; permission conveyed through Copyright Clearance Center, Inc. (**F**) ROS production measured by flow cytometry in MDA-MB-231 cells 24 h after 8 Gy irradiation and according to AGuIX incubation. All data are represented as mean ± standard error of the mean (SEM), with at least 3 independent experiments. Statistical tests were performed using two-tailed *t*-tests. Adapted from Sun et al., 2022 [77]. (**A**–**F**) * *p* < 0.05, ** *p* < 0.01, *** *p* < 0.001.

As previously mentioned, Detappe and coworkers evaluated the influence of a 6 MV-FFF delivery mode on several biological parameters [53]. The study demonstrated that irradiation of AGuIX-incubated tumor cells enhanced ROS production 3 h post-RT as the AGuIX concentration increased (Figure 5A). Moreover, ROS generation was significantly higher with the 6 MV-FFF beam compared to the 6 MV beam. Indeed, the ratio of fluorescence signal with and without AGuIX increased very slightly with increasing AGuIX concentration for the 6 MV beam (from 1 to ~1.05), whereas it significantly increased with the 6 MV-FFF beam, from 1 to ~1.42. These in vitro results highlighted the contribution of kV incoming photons and the increased probability of photoelectric interaction induced by AGuIX, as the FFF beam was associated with a higher amount of low-energy photons.

Then, Simonet and coworkers compared the generation of mitochondrial ROS (mROS, MitoSOX™ reagent, Thermo Fisher Scientific, Figure 5B) and cytosolic ROS (cROS, CM-H_2_DCFDA probe, Thermo Fisher Scientific, Figure 5C) after RT [68]. First, RT did not modify cROS and mROS production in SQ20B cells, whether with or without AGuIX. In fact, SQ20B cells have a high endogenous glutathione (GSH) content, providing radioresistant properties that may explain these results, with GSH being one of the main endogenous cellular ROS regulators. Then, the authors repeated the experiments after GSH depletion and showed that AGuIX induced a significant enhancement of RT-induced cROS and mROS levels immediately after irradiation, most likely related to the consequences of water radiolysis. Indeed, AGuIX significantly increased mROS level normalized on non-treated cells from ~9 to 10 and cROS level from ~75 to 110. Beyond the initial burst, AGuIX continued to increase normalized cROS level at later times, from ~3.5 to 7 after 1 h and from ~5.5 to 12 after 4 h, while mROS level stayed below 2 whatever the time post-RT. Part of the explanation is that AGuIX accumulates in the cytosol but not in the mitochondria, potentially due to the inner membrane impermeability.

Last, three different studies evaluated the influence of AGuIX on ROS production under Cs^137^ γ-rays [48,77,78]. In the first study, Du and coworkers demonstrated that AGuIX increased ROS production in two different tumor cell lines, for at least 6 h (Figure 5D, [48]). Immediately after RT, the total fluorescence intensity significantly increased from ~10,500 with RT alone to ~13,500 with the combined treatment in H1299 cells, and from ~11,000 to 14,500 in A549 cells. Then, 2 and 6 h after RT, the fluorescence intensity stayed below 5000 for all conditions, but the AGuIX addition always resulted in a significant increase in the signal compared to RT alone.

Then, Song et al. evidenced an amplification of RT-induced cellular ROS generation in the presence of AGuIX, up to at least 4 h post-irradiation (Figure 5E [78]). Indeed, adding AGuIX to RT significantly increased the mean total fluorescence intensity from ~1750 to 2000 after 2 h, and from ~1800 to 2100 after 4 h.

Last, Sun and colleagues showed that AGuIX induced a significant increase in ROS production 24 h after irradiation (Figure 5F, [77]). Indeed, the relative ROS content significantly increased from ~2650 to 3200 in MDA-MB-231 cells and from ~2200 to 2650 in MDA-MB-468 cells with the addition of AGuIX to RT.

### 4.4. Conclusions

The five above-mentioned studies were performed with different parameters, including cancer type and cell lines; AGuIX incubation conditions; ROS probe; and RT type, dose, and energy. However, they all demonstrated that AGuIX further increased RT-induced ROS production, sometimes for several hours, showing the versatility of AGuIX nanomedicine.

During AGuIX radiosensitization, ROS are the first species generated after the emission of low-energy electrons following the primary physical interaction. Due to their short half-life, they directly interact with biomolecules in their immediate environment, such as lipids, DNA, and proteins. Moreover, other intracellular targets may be involved in oxidative stress, in particular, lysosomes and mitochondria. Consequently, these biochemical reactions can affect multiple aspects of cell biology and ultimately lead to DNA damage, cell death, and immune activation, which will be discussed later.

## 5. Biological Effects: Any Increase in DNA Damage or Tumor Cell Death?

### 5.1. Mechanisms Affecting DNA

DNA damage production is one of the core mechanisms of RT-induced cell death. As previously mentioned, DNA damage can result from direct DNA ionization or from the reaction of ROS with biomolecules after their generation during water radiolysis [16,28]. However, a large number of DNA damage repair mechanisms constantly limit these genomic aberrations and are a major target of anticancer treatments [2]. Indeed, RT-induced DNA damage can be detected by two appropriate kinases, ataxia–telangiectasia mutated (ATM) and ataxia–telangiectasia and Rad3-related protein (ATR), which will activate DNA damage response (DDR) mechanisms to repair DNA during cell cycle arrest [96]. These repair mechanisms mainly involve homologous recombination (HR) and non-homologous end-joining (NHEJ) pathways [97]. However, when DNA damage is too extensive to be repaired, the cell is unable to maintain its integrity, leading to cell death.

#### 5.1.1. DNA Damage Production

RT can induce different types of DNA damage, including base alterations, DNA–DNA cross-links, SSBs or DSBs, complex damage, and clustered DNA damage [98]. Regarding measurement methods, immunofluorescence (IF) assays are usually used to assess the occurrence of DNA breaks and repair over time by staining the phosphorylated histone γ-H2AX or the DNA-repair protein 53BP1. They are often studied along with fluorescent antibodies to stain foci that correlate with the number of DSBs, but the scoring process is not well standardized yet and may vary between studies. Another widely used method is the Comet assay, which is based on the migration of DNA from the cell nucleus and provides valuable information on DNA alteration after RT. In most cases, tail moment is used as the scoring method, reflecting the extent of DNA damage, although extracting reliable quantitative data can be challenging [90].

When exposed to RT, high-Z elements can absorb more photons and release a high number of secondary particles that can increase DNA damage through the direct or indirect mechanism. To date, several studies showed that high-Z metal-based NPs could increase DNA damage and reduce DNA repair during RT, even when not located in the nucleus [99]. Several in vitro studies investigated DNA damage in the presence of AGuIX, as summarized in Table 4.

**Figure 6:**
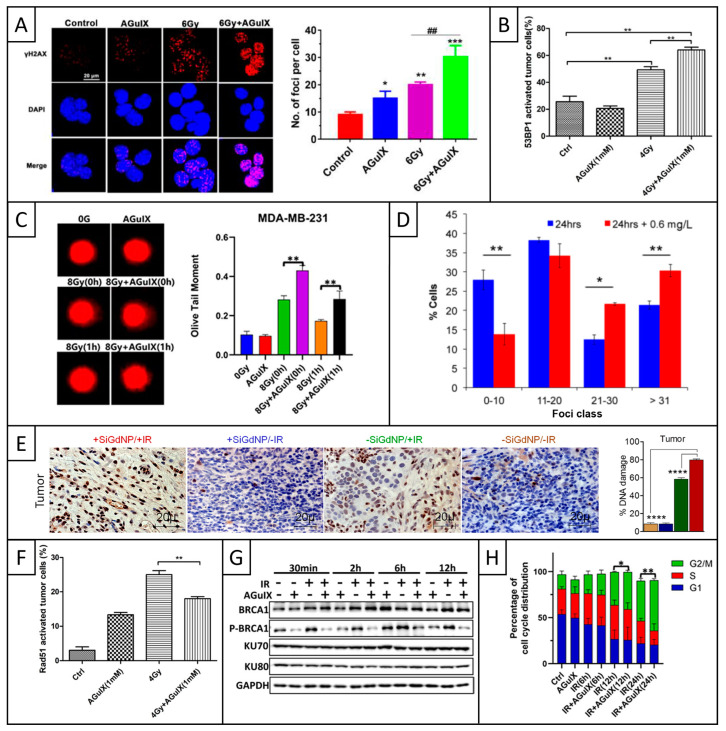
Mechanisms affecting DNA, from DNA damage to cell cycle distribution. (**A**) Fluorescence images representing γ-H2AX foci formation in B16 cells after 6 Gy irradiation and AGuIX incubation (**left**). Scale bar = 20 µm. Quantification of the γ-H2AX foci in each cell, representing DNA damage (**right**). All data are represented as mean ± SD. Statistical tests were performed using one-way analysis of variance (ANOVA) with Tukey statistical hypothesis testing. Used with permission of Royal Society of Chemistry, reprinted from Song et al., 2022 [78]; permission conveyed through Copyright Clearance Center, Inc. (**B**) Quantification of the 53BP1 foci representing DNA strand breaks in H1299 activated cells (>10 foci per cell) after 4 Gy irradiation and AGuIX incubation, 24 h post-irradiation. All data are represented as mean ± SD, with 3 independent experiments. Statistical tests were performed using two-tailed *t*-tests. Reprinted with permission from Du et al., 2020 [48]. Copyright © 2020, American Chemical Society. (**C**) Representative fluorescence images of the comet assay of MDA-MB-231 cells after 8 Gy irradiation and AGuIX incubation, at 30 min (0 h) and 1 h (1 h) post-irradiation (left). Quantification of olive tail moments illustrating DNA strand breaks (right) All data are represented as mean ± SEM, with at least 3 independent replicates. Statistical tests were performed using two-tailed *t*-tests. Reprinted from Sun et al., 2022 [77]. (**D**) Percentage of B16F10 cells in the different γ-H2AX foci class after 2 Gy irradiation and AGuIX incubation, 24 h post-irradiation. Foci classes were defined according to the number of foci per nucleus, for a total of 350 cells counted per condition. All data are represented as mean ± SEM, with 3 independent experiments. Statistical tests were performed using two-tailed unpaired *t*-test. Adapted from Kotb et al., 2016 [51]. (**E**) Tumor tissue of subcutaneous Capan-1 tumor-bearing mice stained with γ-H2AX after 10 Gy irradiation and AGuIX intravenous injection (**left**). γ-H2AX staining was used to show DNA double-strand breaks, with damaged tumor cell nuclei in brown (γ-H2AX+) and viable cells in blue (γ-H2AX−). Magnification: 100×. Scale bar = 20 µm. Quantification of γ-H2AX+ nuclei across multiple image planes (*n* = 50, **right**). Control is in yellow, AGuIX alone in blue, RT alone in green and RT+AGuIX in red. All data are represented as mean ± SD. Statistical tests were performed using two-tailed unpaired *t*-tests. Adapted from Detappe et al., 2016 [52]. Copyright © 2016, with permission from Elsevier. (**F**) Quantification of the RAD51 foci in activated H1299 cells (>10 foci per cell), after 4 Gy irradiation and AGuIX incubation, 24 h after irradiation. All data are represented as mean ± SD, with 3 independent experiments. Statistical tests were performed using two-tailed *t*-tests. Reprinted with permission from Du et al., 2020 [48]. Copyright © 2020, American Chemical Society. (**G**) Results of Western blots realized in MDA-MB-231 cells after 8 Gy irradiation and AGuIX incubation. Expression levels of homologous recombinant repair protein breast cancer 1 (BRCA1) and non-homologous end-linked repair protein (Ku70/80) at the indicated post-irradiation times, + indicates treated cells while – indicates untreated cells, treatment being either RT (IR) or incubation with AGuIX. Reprinted from Sun et al., 2022 [77]. (**H**) Percentage of cell cycle distribution of the MDA-MB-231 cells in the different phases measured by flow cytometry, after γ-ray irradiation and AGuIX incubation. All data are represented as mean ± SEM, with at least 3 independent replicates. Statistical tests were performed using two-tailed *t*-tests. Reprinted from Sun et al., 2022 [77]. * *p* < 0.05, ** *p* or ## *p* < 0.01, *** *p* < 0.001, **** *p* < 0.0001.

In vitro studies evaluated the generation of DNA damage by RT in the presence of AGuIX, assessing the influence of several experimental and irradiation parameters. If all studies agreed on the radiosensitizing effect of AGuIX, they reported various types of DNA damage in cancer cells. Several studies demonstrated that AGuIX increased the proportion of cells with γ-H2AX foci. Indeed, one study showed that the addition of AGuIX increased the number of γ-H2AX foci per cell counted 15 min after RT from ~12 to 24 in H460 cells and from ~10 to 22 in E0771 cells compared to RT alone [101]. At later time points, a study showed that AGuIX induced a significant increase in DNA DSBs 4 h after RT, with ~30 γ-H2AX foci compared to ~20 foci with RT alone (Figure 6A, [78]). Another study demonstrated a significant increase in γ-H2AX-positive cells from ~62 to 72 after 2 h and from ~43 to 58 after 24 h in MDA-MB-231 cells, and similar effects in MDA-MB-468 cells [77]. Last, a study showed that AGuIX significantly increased the proportion of γ-H2AX-positive cells 24 h after RT, from ~45 to 70% in H1299 cells and from ~40 to 55% in A549 cells [48].

Some studies also evaluated the generation of DNA damage with the 53BP1 marker. One study showed that AGuIX increased the proportion of 53BP1-positive cells 30 min after RT compared to RT alone, with ~17 vs. 15 foci per U87 cell and ~23 versus 20 foci per MCF-7 cell [71]. Another study showed that AGuIX significantly increased the proportion of 53BP1-activated cells 24 h after RT, with a rise from ~50 to 65% in H1299 cells (Figure 6B) and from ~40 to 55% in A549 cells [48]. Two other studies evaluated the influence of irradiation parameters: one study showed the influence of the clinical beam energy [53], and the other one showed the role of the radiation dose [52]. Indeed, the first study showed that AGuIX increased the proportion of 53BP1-positive cells compared to RT alone (~35%), and that 6 MV-FFF beam was more efficient compared to standard 6 MV beam (>70% vs. 58%), emphasizing the role of low-energy photons [53]. The other one demonstrated that AGuIX induced a strong increase in the proportion of 53BP1 positive cells under RT, with much more damage at the highest dose of 10 Gy (95.4% vs. 79.4%) compared to 4 Gy (78.1% vs. 60.5%) [52].

Two studies evaluated DNA damage generation both 30 min and 1 h after RT with the Comet assay. First, a study showed that AGuIX increased olive tail moment from ~0.6 to 0.95 after 30 min and from ~0.3 to 0.75 after 1 h in H1299 cells; and from ~0.38 to 0.46 after 30 min and from ~0.32 to 0.4 after 1 h in A549 cells. The additional effect of AGuIX was still observable at 24 h, but it was not so visible, as almost all DNA damage was already repaired [48]. Another study showed a significant increase in olive tail moment, from ~0.27 to 0.42 after 30 min and from ~0.18 to 0.28 after 1 h in MDA-MB-231 cells (Figure 6C), and similar effects were seen in MDA-MB-468 cells. This study also reported more DNA fragmentation damage in the group containing AGuIX [77].

As results of DNA damage measurements are critically impacted by the delay between irradiation and the experiment, some studies focused on the kinetics of DNA damage generation in the presence of AGuIX. Several studies showed that AGuIX was able to rapidly increase RT-induced DNA damage in the first hour following irradiation [48,71,77,101]. Then, some studies evaluated the generation of DNA damage in the first 24 h post-RT and demonstrated that AGuIX was also able to increase late DNA damage [48,51,68,77,78,100]. Consequently, different in vitro studies demonstrated the ability of AGuIX to increase RT-induced DNA damage at early stages post-RT, as well as at later times.

Last, a few studies demonstrated the ability of AGuIX to induce the generation of complex DNA lesions and prevent their repair. Indeed, several studies showed the capacity of AGuIX to increase the proportion of cells with a large number of foci 24 h after RT, compared to cells only irradiated [51,68,100]. Indeed, according to the study, the proportion of cells with a high number of foci increased from ~34 to 52% (>20, Figure 6D, [51]); from less than 5% to ~20% and 40% with carbon ions and X-rays, respectively (>15, [100]); and from 23 to 45 (>50, [68]).

In addition to DNA damage, AGuIX also increased the accumulation of RT-induced micronuclei in the cytosol of cancer cells, thus increasing their genomic instability [102]. Micronuclei are cytosolic events frequently associated with nuclear membrane alterations and occurring after the induction of DNA DSBs. More precisely, AGuIX increased the proportion of cells with micronuclei from ~16% to 26 and 27% at respective concentrations of 0.6 and 1.2 mM in Caco-2 cells, and from ~24% to 34 and 41% in CT26 cells. Regarding the mechanisms of action, AGuIX was shown to trigger cell cycle progression through mitosis, as well as ROS generation, which are both involved in micronuclei formation. Moreover, AGuIX promoted cyclic guanosine monophosphate–adenosine monophosphate synthase (cGAS) recruitment to micronuclei, enhanced the secretion of type I interferon (IFN), and stimulated the RT-driven cGAS/STING/type I IFN signaling pathway (STING: stimulatory IFN genes). Therefore, these studies revealed that AGuIX induced more complex and lethal damage compared to RT alone.

Some studies also observed no impact of AGuIX on the generation of DNA damage. First, a study showed that AGuIX did not modify the proportion of γ-H2AX-positive cells 24 h after RT [102]. This result may be explained by the fact that almost all the cells presented γ-H2AX foci in the RT-alone group, maybe due to the sensitivity of Caco-2 cells. Interestingly, this study also revealed a significant increase in the tail moment with AGuIX, which will be discussed below. Another study demonstrated no significant influence of AGuIX on DSBs, either by γ-H2AX or by 53BP1 markers, at observation times ranging from 5 min to 8 h at 1 Gy and from 1 to 24 h at 4 Gy after γ-ray irradiation [67]. The authors concluded that AGuIX did not enter the nucleus even after a long incubation time and did not directly affect nuclear DNA. Interestingly, they hypothesized that AGuIX radiosensitization observed in clonogenic assays was triggered by damage to the lysosomes and endosomes, key targets previously discussed. Moreover, the apparent absence of specific DNA damage could be explained by experimental conditions, including suboptimal concentration or the nature of ^60^Co γ-ray irradiation. Last, a study used the Comet assay to study SSBs formation and observed no difference in the presence of AGuIX [68]. In addition to the nature of the observed damage, one possible explanation is the radioresistant nature of the SQ20B cell line, which is characterized by high levels of GSH that may scavenge ROS.

A few in vivo studies also investigated DNA damage under RT in the presence of AGuIX, as presented in Table 5, with γ-H2AX staining of tumor and normal tissue.

Three studies demonstrated that AGuIX increased the proportion of RT-induced DNA damage in tumor tissue, either 30 min [52,53] or 1 h after RT [48]. Indeed, a first study demonstrated an increase in DNA damage from ~60% to more than 80% (Figure 6E, [52]). Another study showed much higher damage when RT was delivered with 6 MV-FFF (78%) compared to 6 MV (36%) in the combined-treatment groups, confirming the major contribution of low energy photons previously mentioned in these conditions [53]. A last study reported a significant increase in DNA damage from ~43% to 58% [48]. In all the above-mentioned studies, no additional DNA damage was attributed to AGuIX in healthy organs, even for those located close to the irradiation site, confirming the safety of the nanomedicine [48,52,53].

#### 5.1.2. DNA Repair Mechanisms

Beyond the capacity of AGuIX to increase RT-induced DNA damage, several studies investigated its role in the different repair mechanisms of this damage. First, two studies compared γ-H2AX foci 30 min and 24 h after RT and highlighted the implication of AGuIX in the DNA damage repair process by showing stronger foci disappearance after 24 h in the RT alone groups [51,100]. Indeed, AGuIX addition significantly mitigated the decrease in the number of foci per cell after 24 h, which was 19.3 for RT alone and 28 for the combined treatment, corresponding to a 45% enhancement in DSBs [51]. Similarly, cells incubated with AGuIX presented a significantly higher number of γ-H2AX foci after 24 h compared to cells that were only irradiated, whatever the irradiation type that was either carbon ions (14 vs. 8 foci/cell) or X-rays (10 vs. 6 foci/cell) [100].

Two other studies elucidated some of the signaling pathways involved in DNA damage repair in the presence of AGuIX [48,77]. First, Du et al. evaluated HR repair by studying the colocalization of γ-H2AX foci and the RAD51 recombinase, a key element of this repair pathway [48]. The results highlighted a significant reduction in RAD51 recruitment to the DNA damage regions in the cells treated with AGuIX, confirming its capacity of reducing HR repair under RT (Figure 6F). This was further confirmed by the decrease in breast cancer 1 (BRCA1) expression level, a key protein that mediates HR repair and may have some antagonistic effect on NHEJ. The authors also highlighted that HR repair consisted of several stages in the hours following RT, with an initial increase in DDR capacity, followed by a reduction in long-term repair capacity. Indeed, AGuIX first activated the MRN-ATM-Chk2 pathway 30 min after RT, before inhibiting it after 2 h. Last, the results demonstrated a mild increase in Ku70 and Ku80 levels in the presence of AGuIX, as well as p-DNA-PK, indicating a slight increase in NHEJ.

Then, Sun and coworkers studied the role of AGuIX in the DNA damage repair process by measuring the expression of several proteins involved in the repair pathway using Western blots [77]. These proteins included the MRN complex, ATM, p-ATM, p-Chk2, p53/p-p53, BRCA1 (HR), and Ku70/80 (NHEJ). The results demonstrated that AGuIX may increase RT-induced DNA damage by reducing the HR repair ability. Indeed, AGuIX inhibited the expression of some DDR proteins, including BRCA1 (Figure 6G), and had a strong inhibitory effect on the MRN-ATM-Chk2 signaling pathway. Moreover, unlike the previous study, AGuIX does not seem to involve the NHEJ pathway, as no change in the expression level of the Ku70 and Ku80 proteins was observed.

Altogether, these results show that AGuIX further increases RT-induced DNA damage by inhibiting DNA repair ability, mainly acting through the HR pathway. These results provide some clues to explain the significant impact of AGuIX at later time points, due to HR repair inhibition.

#### 5.1.3. Cell Cycle and DNA Replication

The cell cycle is a process that leads to DNA replication and cell division, consisting in four distinct phases. First, the Gap 1 (G1) phase prepares for DNA replication, which occurs during the synthesis (S) phase. Then, the Gap 2 (G2) phase controls the duplicated DNA for errors and prepares for division, which takes place during the mitosis (M) phase. Cell cycle progression is regulated by various checkpoints that could induce cell cycle arrest in the case of cellular DNA damage. These checkpoints are controlled by regulatory proteins, mainly ATM, Chk2, and P53 for the G1/S transition; and ATR and Chk1 for S and G2/M phase progression [48,77]. All of these regulatory processes help maintain genomic integrity and prevent uncontrolled division.

Beyond DNA damage and repair, several studies investigated the impact of AGuIX on the distribution of the cells in the different phases of the cell cycle, as presented in Table 6.

Two studies demonstrated that AGuIX significantly increased the proportion of cells in the G2 phase 24 h after RT, and that this percentage increased in the hours following RT [48,77]. Indeed, a first study showed that the proportion of cells in the G2 phase increased over time post-irradiation (from 6 to 24 h), and that after 24 h, it increased from ~18 to 27% at 4 Gy and from ~30 to 45% at 8 Gy [48]. Similarly, another study showed a significant increase in the G2/M phase after 24 h, from ~45 to 55% in MDA-MB-231 cells (Figure 6H) and from ~40 to 50% in MDA-MB-468 cells [77]. As previously discussed regarding DNA damage, AGuIX also inhibited the MRN-ATM-Chk2 signaling pathway, attenuating G1/S cell cycle arrest [77]. Moreover, they also stimulated the phosphorylation of ATR and Chk1, probably explaining G2/M cell cycle arrest. Thus, AGuIX is able to stop the progression of cells through the cell cycle phases to prevent cell division of irradiated tumor cells.

#### 5.1.4. Conclusions

After discussing the role of AGuIX in radiation–matter interactions and oxidative stress, this review considered its influence on DNA damage. Most in vitro studies demonstrated the ability of AGuIX to increase RT-induced genotoxicity, either immediately or several hours after RT depending on the experimental conditions. A few studies also emphasized the impact of AGuIX on the increase in complex DNA lesions and micronuclei formation. Moreover, AGuIX stimulates the cGAS/STING/type I IFN signaling pathway and may support the development of an effective antitumor immune response, which will be discussed hereafter. In terms of mechanisms, AGuIX further increases the degree of RT-induced DNA damage by weakening DNA damage repair pathways, in particular, the HR repair pathway. Moreover, a few studies demonstrated the capacity of AGuIX to induce G2/M phase arrest, which limits the propagation of damaged DNA and uncontrolled cell division. Regarding in vivo studies, they highlighted the role of AGuIX in increasing RT-induced DNA damage to tumor tissue only, demonstrating the pharmacological specificity and performance of the nanomedicine.

Beyond these statements, several studies tried to elucidate some of the biological mechanisms leading to tumor cell death, at the tumor cell level itself but also through an action on the TME.

### 5.2. Mechanisms of Cell Death and Immune Response

Beyond the chemical and biochemical events previously discussed, RT is also able to induce various types of cell death, including apoptosis, necrosis, autophagic cell death, ferroptosis, and immunogenic cell death (ICD). Numerous studies evaluated the impact of AGuIX on RT-induced cell death mechanisms, as presented hereafter.

#### 5.2.1. Apoptosis

Apoptosis is a highly regulated process mainly recognizable by cell shrinkage, pyknosis, and intact cell membrane. On the opposite, necrosis is an unregulated mechanism characterized by cell swelling, disrupted cell membrane, and cytoplasm released [96,103]. Numerous experiments focused on the identification of apoptosis versus necrosis as a consequence of combining AGuIX with RT, either in vitro, as summarized Table 7, or in vivo, as presented in Table 8.

To evaluate apoptosis, many of these studies performed flow cytometry experiments by using Annexin V-FITC in combination with a cell-viability dye. This double staining allows the distinction between late apoptotic cells (positive for both dyes), early apoptotic cells (Annexin V-positive and viability dye-negative), and necrotic cells (Annexin V-negative and viability dye-positive).

**Figure 7:**
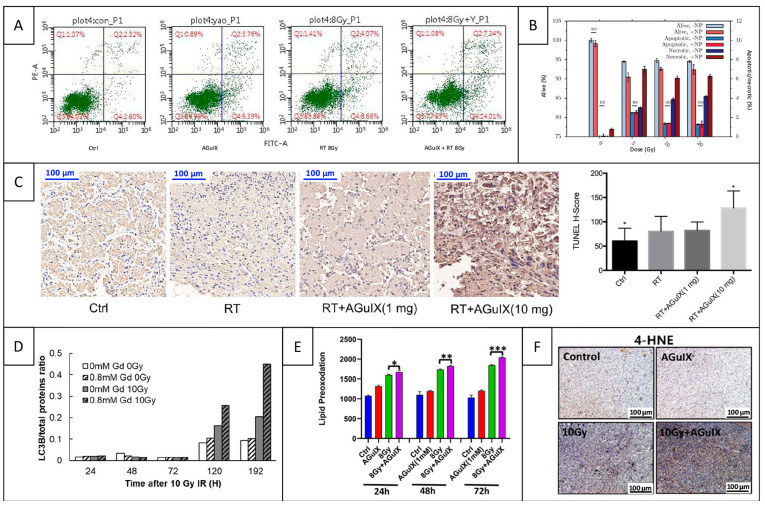
Several types of cell death mechanisms. (**A**) Representative flow cytometry data plots showing the proportion of H1299 cells in early and late apoptosis, as well as necrosis after 8 Gy irradiation and incubation with AGuIX, 24 h post-irradiation. Adapted with permission from Du et al., 2020 [48]. Copyright © 2020, American Chemical Society. (**B**) Percentage of apoptotic and necrotic F98 cells measured by flow cytometry as a function of the received irradiation dose (from 0 to 20 Gy) and in the absence (−NP) or presence (+NP) of AGuIX. Control cells were considered to have 100% of living cells and 0% of both apoptotic and necrotic subpopulations. The values obtained with the different treatments were normalized on the control cell group. All data are represented as mean ± SEM. Statistical tests were performed using standard two-tailed ANOVA. If there are non-significant differences with *p* > 0.05, they are indicated on the graphs as “ns”. Used with permission of Royal Society of Chemistry, adapted from Yousef et al., 2016 [76]; permission conveyed through Copyright Clearance Center, Inc. (**C**) Tumor tissue of subcutaneous HepG2 tumor-bearing mice stained with terminal deoxynucleotidyl transferase dUTP nick end labeling (TUNEL) after 6 Gy irradiation and AGuIX intravenous injection (left). Immunohistochemistry (IHC) was used to study apoptosis, with viable nuclei stained in blue and apoptotic ones stained in brown. Magnification: 200×. Scale bar = 100 µm. Quantification of TUNEL staining in tumor tissue using the histochemistry score (H-Score, right). All data are represented as mean ± SD. Statistical tests were performed using Student’s *t*-test. Adapted from Hu et al., 2019 [54]. (**D**) Ratio of microtubule-associated protein 1 light-chain 3B (LC3B) to total proteins in SQ20B cells exposed to 10 Gy irradiation and AGuIX incubation. Quantification was based on Western blot results, and LC3B was used as a specific autophagosome antibody to study autophagy. Data are representative of 2 independent experiments. Used with permission of American Scientific Publishers, reprinted from Simonet et al., 2020 [68]; permission conveyed through Copyright Clearance Center, Inc. (**E**) Lipid peroxidation levels of the MDA-MB-231 cells after an 8 Gy irradiation and AGuIX incubation. Results were obtained by flow cytometry using the boron-dipyrromethene (BODIPY) 581/591 C11 dye. All data are represented as mean ± SEM, with at least 3 independent replicates. Statistical tests were performed using two-tailed *t*-tests. Reprinted from Sun et al., 2022 [77]. (**F**) Tumor tissue of subcutaneous MDA-MB-231 tumor-bearing mice stained with 4-hydroxynonenal (4-HNE) after 10 Gy irradiation and AGuIX intravenous injection. IHC was used to study ferroptosis; strong 4-HNE staining being a sign of high lipid peroxidation, suggesting ferroptosis. Scale bar = 100 µm. Adapted from Sun et al., 2022 [77]. * *p* < 0.05, ** *p* < 0.01, *** *p* < 0.001.

Among these studies, a large number demonstrated that AGuIX increased cell apoptosis but not cell necrosis under RT [48,77,78]. It has been shown that AGuIX significantly increased both RT-induced early and late apoptosis after 24 h in two different cell lines (Figure 7A, [48]). Indeed, after 8 Gy irradiation, early cell apoptosis increased from ~5 to 10% in H1299 cells and from ~9 to 20% in A549 cells, and late apoptosis from ~2 to 9% in H1299 cells and from ~6 to 7% in A549 cells in the presence of AGuIX. The authors also found that AGuIX action probably involves regulation of caspase-3 and cellular inhibitors of apoptosis protein 1 levels. Another study reported similar results and demonstrated that AGuIX induced a significant increase in both early and late apoptosis after 24 and 48 h in two other different cell lines compared to RT alone [77]. Indeed, 48 h after an 8 Gy irradiation, total cell apoptosis increased from ~6 to 8% in MDA-MB-231 cells, and from ~7.5 to 10.5% in MDA-MB-468 cells in the presence of AGuIX. Regarding markers of cell apoptosis, Western blots revealed a rise in expression level of cleaved caspase-3 and cleaved PARP, a DNA repair enzyme that promotes cell disintegration after cleavage. This study thus hypothesized that AGuIX increased apoptosis by activating caspase-3 and PARP. Last, AGuIX induced more morphological alterations due to apoptosis as cell shrinkage and vesicles on cell membranes. In contrast, another study showed that AGuIX only increased RT-induced late apoptosis, both 6 h after RT, with a proportion of cells from 3.9 to 8.26%, and 24 h after RT, with an even greater increase from 4.1% to 16.9% [78].

It is worth mentioning that some authors found that the difference in cell apoptosis level under RT was not significant with or without AGuIX [52]. Indeed, 15 min after 4 Gy-RT, 41.8% of cells were in early apoptosis and 23% in late apoptosis with the combined treatment, compared to 37.6% and 21.9% with RT alone, respectively. The trend stayed the same after 24 and 48 h, with a slight decrease in total apoptosis.

Last, a single study demonstrated that AGuIX was able to significantly increase the level of necrotic cells under RT [76]. Indeed, 24 h after a 5 Gy irradiation, while the proportion of apoptotic cells stayed below 3% in both conditions, the proportion of necrotic cells increased from 3 to 7% in the presence of AGuIX (Figure 7B).

A few in vivo studies also evaluated cell apoptosis by using terminal deoxynucleotidyl transferase dUTP nick end labeling (TUNEL) staining in tumor tissue. Three studies reported that AGuIX significantly increased the proportion of RT-induced apoptotic cells, rising from ~12 to 16% [48], from ~11 to 18% [77], and from ~15 to 35% [49]. Another study confirmed the above-mentioned results with a visual increase in TUNEL-positive cells observed in tumor slices for the combined-treatment group compared to RT alone [78]. At last, the comparison between two doses of AGuIX (1 and 10 mg) on cell apoptosis was evaluated by two different methods [54]. The authors determined the histochemistry score (H-SCORE) based on TUNEL staining, corresponding to the sum of the percentage of cells with a certain intensity multiplied by that intensity, with intensities rated from 1 (low) to 3 (strong). The results showed that AGuIX induced a significant increase in H-SCORE at the highest dose only, from ~80 for RT alone and RT + AGuIX (1 mg) groups to ~130 for the RT + AGuIX group at 10 mg (Figure 7C). These experiments were supplemented by single-photon emission computed tomography (SPECT/CT) imaging with the ^99m^Tc-duramycin apoptosis agent that confirmed immunohistochemistry (IHC) results. Indeed, the tumor-to-background ratio significantly increased from around 1 for the first three groups (control, RT alone, and combined treatment at 1 mg) to 3.87 for the combined-treatment group at 10 mg, demonstrating a high degree of cell apoptosis. In brief, AGuIX is able to increase RT-induced cell death both in vitro and in vivo by activating some effectors of the caspase family and inducing both early and late apoptosis.

#### 5.2.2. Autophagy

Autophagy is a highly regulated process that is characterized by the sequestration of certain cytoplasmic organelles within vesicles for further lysosomal degradation in reaction to stress factors [96,103]. It plays a dual role in the regulation of tumor cell death, as it can promote cell survival under stressful conditions but also lead to various forms of cell death when it becomes excessive, including autophagic cell death, apoptosis, or ICD [104].

One study found that AGuIX was not associated with an increase in cell apoptosis assessed through caspase activation, nor in necrosis with the Annexin V/PI assay, but was indeed able to induce autophagic cell death [68]. SQ20B cells were incubated with AGuIX for 24 h at 0.8 mM, exposed to X-rays (10 Gy, 250 kV). The authors focused on the autophagosome and demonstrated a strong increase in the expression of microtubule-associated protein 1 light-chain 3B (LC3B), as measured by Western blot analysis, when the cells were incubated with AGuIX before RT compared to RT alone (Figure 7D). Regarding the kinetics, the ratio of LC3B to total proteins stayed below 0.05 until 72 h after 10 Gy irradiation and increased from ~0.16 to 0.26 after 120 h, and from ~0.21 to 0.45 after 192 h. The results also demonstrated that autophagy was involved in the radiosensitizing effect of AGuIX and may mediate the combination therapy efficacy. Last, the study revealed potential signs of autosis, an autophagy-dependent form of cell death, and further experiments are required to decipher the involved mechanisms.

#### 5.2.3. Ferroptosis

Ferroptosis is another form of regulated cell death that is iron-dependent and characterized by lipid peroxidation and excessive ROS accumulation in cells. Since the observed DNA damage and cell apoptosis were not sufficient to explain the level of radiosensitization provided by AGuIX, Sun and colleagues decided to evaluate the induction of ferroptosis on MDA-MB-231 and MDA-MB-468 cells [77]. Cells were previously incubated with AGuIX for 1 h at 1 mM and irradiated with Cs^137^ γ-rays (8 Gy, 662 keV). First, TEM was used to study the ultrastructure of MDA-MB-231 cells 24 h after RT: the images revealed more obvious morphological changes in the mitochondria with the combined-treatment group compared to RT alone. Two methods were then used to study lipid peroxidation, including flow cytometry analysis of boron-dipyrromethene (BODIPY) 581/591 C11 and Malondialdehyde (MDA) sensors. First, the AGuIX effect increased over time after RT, from 24 h to 72 h, with a significant increase in lipid peroxidation of ~10% in MDA-MB-231 cells (Figure 7E) and of ~15% in MDA-MB-468 cells after 72 h. Moreover, AGuIX addition significantly increased MDA concentration by ~29% in MDA-MB-231 cells and by ~8% in MDA-MB-468 cells. Then, the authors performed clonogenic assays with a ferroptosis inhibitor called ferrostatin-1 and demonstrated that its addition mitigated part of the inhibitory effects of AGuIX. Indeed, adding ferrostatin-1 reduced the radiosensitizing ratio of MDA-MB-231 cells from 1.803 to 1.315 and that of MDA-MB-268 cells from 1.306 to 1.167, suggesting that AGuIX radiosensitization partly involves ferroptosis. Last, the same authors also evaluated ferroptosis in vivo by assessing the level of 4-hydroxynonenal (4-HNE) in tumor tissues by IHC. The images showed that AGuIX combined with RT further increased the expression of 4-HNE, an aldehyde product of lipid peroxidation, compared to RT alone (Figure 7F).

By focusing on the underlying mechanisms, the study demonstrated that AGuIX did not affect the metabolism of normal iron and fatty acid, two factors involved in ferroptosis. However, AGuIX was able to inhibit the nuclear factor erythroid 2-related factor 2 (NRF2), which can activate the cysteine transport system to promote GSH synthesis during ferroptosis. AGuIX also inhibited the cysteine transport system, reducing ferroptosis resistance. Regarding GSH, AGuIX further decreased its content in the two cell lines compared to RT alone, and this excessive consumption of intracellular GSH may be involved in the antitumor effect of AGuIX. Last, AGuIX impacted the activity of GSH peroxidase 4 (GPX4), a key regulator of ferroptosis. Indeed, GPX4 protein was upregulated at 6 h and 12 h post-RT, demonstrating an adaptive response to ferroptosis in the cells. Then, GPX4 level decreased at 24 and 48 h after RT, suggesting that AGuIX was able to weaken the resistance to ferroptosis at this time.

To summarize, AGuIX reduces the resistance of tumor cells to the regulated cell death process called ferroptosis. Initial lipid peroxidation causing an oxidative stress leads to morphological changes in mitochondria and then to the inhibition of the NRF2-GSH-GPX4 signaling pathway after irradiation.

#### 5.2.4. ICD and Immune Response

ICD is a specific type of regulated cell death that occurs within the TME following RT irradiation. RT not only induces the death of cancer cells but also triggers the release of damage-associated molecular patterns (DAMPs), which activate the immune system and enhance the antitumor immune response. Among these DAMPs, high-mobility group box 1 (HMGB1) and calreticulin (CRT) play crucial roles. HMGB1 is released from dying cells and contributes to the activation of cytotoxic T cells by stimulating dendritic cells (DCs) and promoting an inflammatory response [105]. On the other hand, CRT is exposed on the surface of apoptotic cells, acting as a signal for phagocytosis by DCs and increasing adaptive immunity by activating T cells, further amplifying the antitumor immune response [106]. Thus, RT not only destroys tumor cells but also plays a key role in immunomodulation by inducing ICD mechanisms and triggering a protective immune response.

RT also induces the activation of the cGAS-STING pathway in both tumor and DCs [107]. This activation leads to the release of cytokines that subsequently amplifies the immune response by promoting the recruitment of immune cells to the TME, including cytotoxic T cells. Moreover, RT can also trigger the reprogramming of tumor-associated macrophages (TAMs) toward a pro-inflammatory phenotype, further amplifying ICD-induced immune activation [108].

Finally, RT can be appropriately combined with immune checkpoint inhibitors (ICIs) to restore T cell-mediated antitumor immunity. Indeed, the action of cytotoxic T cells often becomes dysfunctional during tumor progression, resulting in limiting efficacy in eradicating tumor cells. Moreover, under specific conditions, the immune response can extend systemically and allows activated immune cells to recognize and eliminate tumor cells at distant sites, thereby contributing to the abscopal effect [109]. Thus, the ability of RT to stimulate both local and systemic antitumor immune response can be amplified when combined with ICIs such as anti-programmed cell death protein 1 (anti-PD-1 or α-PD-1), demonstrating its promise in cancer immunotherapy [110,111].

**Figure 8:**
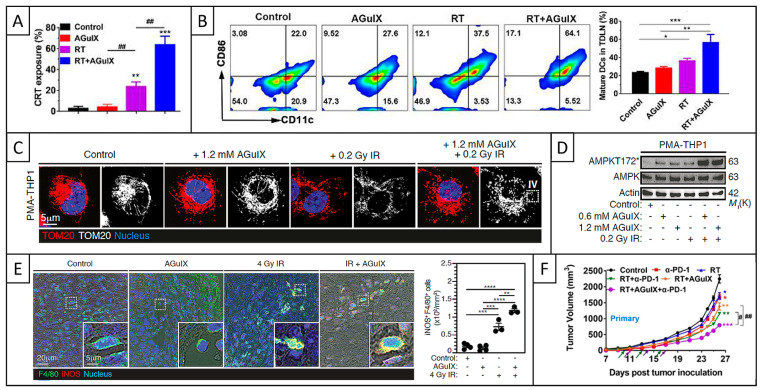
Stimulation of immune response. (**A**) Percentage of calreticulin (CRT) exposure in tumor tissue of subcutaneous B16 tumor-bearing mice after 4 Gy irradiation and AGuIX intravenous injection. CRT expression stained for immunofluorescence (IF) was used to evaluate immunogenic cell death (ICD). All data are represented as mean ± SD. Statistical tests were performed using one-way ANOVA with Tukey statistical hypothesis testing. Used with permission of Royal Society of Chemistry, reprinted from Song et al., 2022 [78]; permission conveyed through Copyright Clearance Center, Inc. (**B**) Representative flow cytometry data plots showing the proportion of CD11c^+^ and CD86^+^ dendritic cells (DCs) in tumor-draining lymph nodes (TDLNs) of subcutaneous B16 tumor-bearing mice after irradiation and AGuIX intravenous injection (**left**). Percentage of mature DCs (CD11c^+^CD86^+^) in TDLNs (**right**). All data are represented as mean ± SD, with at least 3 independent replicates. Statistical tests were performed using one-way ANOVA with Tukey statistical hypothesis testing. Used with permission of Royal Society of Chemistry, adapted from Song et al., 2022 [78], permission conveyed through Copyright Clearance Center, Inc. (**C**) Confocal micrographs of mitochondrial networks of phorbol-12-myristate 13-acetate (PMA) differentiated human THP1 macrophages stained for the translocase of the outer mitochondrial membrane 20 (TOM20, red or white) after 0.2 Gy irradiation and AGuIX incubation. Nuclei are stained in blue. Scale bar = 5 µm. Data are representative of *n* = 3 independent experiments. Reprinted from Muradova et al., 2024 [112]. (**D**) Results of Western blots realized in PMA-differentiated THP1 macrophages after 0.2 Gy irradiation and AGuIX incubation. Expression levels of the adenosine monophosphate-activated protein kinase (AMPK) and phosphorylation of AMPK on threonine 172 (AMPKT172*), + indicates treated cells while − indicates untreated cells, treatment being either RT (IR) or incubation with AGuIX at two different concentrations. Actin is used as loading control. Data are representative of *n* = 3 independent experiments. Reprinted from Muradova et al., 2024 [112]. (**E**) Confocal micrographs of tumor-associated macrophages (TAMs) detected on tumor biopsies from CT26 tumor-bearing mice obtained 19 days after 4 Gy irradiation or AGuIX intravenous injection (**left**). TAMs are stained for inducible nitric oxide synthase (iNOS, red) and F4/80 (green), and nuclei are stained in blue. Scale bar =20 µm. Squares indicate regions of interest (ROIs) for magnification, and inserts show magnifications of selected ROIs, scale bar = 5 µm. Counts of pro-inflammatory TAMs (iNOS^+^F4/80^+^, **right**). All data are represented as mean ± SEM, with at least 3 independent replicates. Statistical tests were performed using one-way ANOVA with Tukey statistical hypothesis testing. Reprinted from Muradova et al., 2024 [112]. (**F**) Evolution of primary tumor volume over time post tumor induction, in subcutaneous B16 tumor-bearing mice. Mice were treated by 4 Gy irradiation, AGuIX intravenous injection and/or α-programmed cell death protein 1 (α-PD-1) intraperitoneal injection. All data are represented as mean ± SD, with 5 to 10 animals per condition. Statistical tests were performed using one-way ANOVA with Tukey statistical hypothesis testing. Used with permission of Royal Society of Chemistry, reprinted from Song et al., 2022 [78]; permission conveyed through Copyright Clearance Center, Inc. * *p* or # *p* < 0.05, ** *p* or ## *p* < 0.01, *** *p* < 0.001, **** *p* < 0.0001.

Several studies investigated the influence of AGuIX in the above-mentioned RT-induced immune mechanisms. Regarding ICD, one study focused on the release of DAMPs in B16 tumor cells that were pre-incubated with AGuIX for 1 h at a concentration of 1 mM and subsequently irradiated with Cs^137^ γ-rays (6 Gy, 662 keV) [78]. The experiments demonstrated that AGuIX increased CRT cell membrane exposure (Figure 8A) and induced higher levels of HMGB1 compared to RT alone with a significant increase from ~2 to 13 ng/mL. This observation is consistent with aforementioned results showing the capacity of AGuIX to increase RT-induced autophagy, as HMGB1 protein is also known to promote this type of cell death [104]. ICD induction was also investigated in vivo in B16 tumor-bearing mice [78], and the results showed that AGuIX increased CRT expression in tumor tissue as well, with an increase from ~24 to 64% compared to RT alone. Thus, AGuIX is able to increase RT-induced ICD through the release of DAMPs, including CRT and HMGB1.

While most of the studies evaluating the effects of AGuIX primarily focused on tumor cells, recent research investigated its potential to activate immune cells within the TME. Indeed, a preliminary investigation focused on AGuIX’s ability to activate the immune system in the absence of RT by specifically evaluating the molecular and functional responses of human natural killer (NK) and pan T cells [113]. These in vitro experiments were performed using NK or pan T cells isolated from peripheral blood mononuclear cells (PBMC), with AGuIX incubated for 48 h at 150 µg/mL. The study first demonstrated that AGuIX alone exhibited no apparent toxicity toward NK or pan T cells. Then, the experiments assessed RNA expression focusing on activation and function associated genes and demonstrated that AGuIX behaved predominantly like control, with little impact on gene expression. Thus, the results showed that AGuIX alone (i.e., without RT) is insufficient to induce transcriptional changes in NK and pan T cells, suggesting an absence of immune cell activation, ICD, and subsequent immune stimulation.

Another study investigated the potential of AGuIX to stimulate an immune response in combination with RT, specifically on CD8^+^ T cells [102]. First, the authors highlighted the importance of the radiation dose in the combined treatment, as the combination of 8 Gy with AGuIX was ineffective while AGuIX increased the 4 Gy-induced antitumor immune response. Indeed, the experiments performed in CT26 tumor-bearing mice showed that AGuIX increased the 4 Gy-induced infiltration of CD8^+^ T cells in tumors from ~14% to 16%. The study also showed that the antitumor immune response triggered by AGuIX and RT was heavily dependent on CD8^+^ T-cells, as depleting CD8^+^ T-cells with anti-CD8 antibodies eliminated the combined treatment’s efficacy, both in terms of survival and tumor volume.

Moreover, another study evaluated the effects of AGuIX on DCs, by analyzing DC population in tumor-draining lymph nodes (TDLNs) of B16 tumor-bearing mice [78]. Flow cytometry experiments revealed that AGuIX increased the proportion of mature DCs (CD11c^+^CD86^+^) in the TDLNs from ~37% to 57% compared to RT alone (9.9B), highlighting its role in promoting DCs’ activation and maturation in vivo, thereby facilitating the recruitment of CD8^+^ T cells.

Last, other researchers explored the ability of AGuIX to reprogram TAMs, particularly in combination with RT [112]. Interestingly, AGuIX was able to trigger the same signaling pathway as RT, but the combination of both treatments resulted in a stronger effect. RT has been shown to induce phenotypic changes in TAMs through a signaling pathway involving NADPH oxidase 2 (NOX2) activation, ROS production, and DNA damage [108]. These steps trigger a DDR and the phosphorylation of the ATM kinase, which regulates macrophage phenotypic conversion via IFN regulatory factor 5 (IRF5) expression. The combination of RT and AGuIX was evaluated on both murine and human macrophages, with an AGuIX incubation for 1 to 48 h at concentrations ranging from 100 nM to 200 nM and a radiation dose of 0.2 Gy. The combined treatment resulted in an increase in γ-H2AX foci, ATM phosphorylation, and the upregulation of pro-inflammatory biomarkers, including inducible nitric oxide synthase (iNOS), IRF5, and cytokines interleukins IL-1β and IL-6. Additionally, the authors observed an increase in mitochondrial fragmentation in human macrophages, as indicated by the expression of the translocase of the outer mitochondrial membrane 20 (TOM20) (Figure 8C). The signaling pathway associated with this fragmentation involved a strong phosphorylation of the adenosine monophosphate activated protein kinase (AMPK) on threonine 172 (AMPKT172*, Figure 8D). These results were further confirmed by the analysis of tumor biopsies from subcutaneous CT26 tumor-bearing mice that evidenced an increase in AMPKT172*^+^ and pro-inflammatory iNOS^+^ TAMs (Figure 8E) with the combined treatment compared to RT alone. Overall, these findings demonstrate the synergistic effect of AGuIX and RT in promoting the reprogramming of TAMs toward a pro-inflammatory phenotype. It further highlights AGuIX’s influence on the TME and its role in stimulating an antitumor immune response.

In brief, the above-mentioned studies evidenced that AGuIX was able to increase RT-induced ICD, contributing to the activation of immune cells such as T cells, DCs and M1 TAMs and thereby inducing the elimination of tumor cells. Beyond AGuIX’s capacity to stimulate a systemic antitumor immune response, two studies prompted further exploration into its potential synergy with ICIs, such as the α-PD-1 antibody. α-PD-1 blocks the interaction between the PD-1 receptor and its PD-L1 ligand, enabling T cells to mount an effective antitumor immune response that results in cancer cell destruction.

A first study showed that the combination treatment of AGuIX with 4 Gy-RT and α-PD-1 significantly reduced tumor volume in B16 tumor-bearing mice compared to all other conditions (Figure 8F, [78]). The study also evidenced that the combination therapy significantly increased the proportion of both CD4^+^ and CD8^+^ T cells in mice lymph nodes and decreased the number of immunosuppressive cells in tumor tissues, including regulatory T cells (Tregs) and myeloid-derived suppressor cells (MDSCs). Moreover, the authors used a bilateral subcutaneous tumor model in which they only irradiated the primary tumors to evaluate a potential systemic antitumor effect. They evidenced an abscopal effect by showing that the combined treatment also reduced the growth of distant tumors that had not been irradiated, evidencing an effective systemic antitumor immune response. Thus, AGuIX could be successfully combined with RT and checkpoint blocking therapy, as the combination therapy is able to activate a systemic immune response and to reverse the immunosuppressive environment.

Another study evaluated the same triple combination therapy, which added no benefit in tumors sensitive to anti-PD-1 therapy, but significantly improved outcomes in animals with anti-PD-1-resistant tumors [102]. Indeed, subcutaneous CT26 tumor-bearing mice treated with AGuIX combined with 4 Gy-RT and anti-PD-1 presented decreased tumor volume and better survival compared to all other treatment groups, with 55% complete remission compared to 22% with RT and anti-PD-1, 11% with RT and AGuIX, and 0% with anti-PD-1 alone or with AGuIX. Thus, these experiments demonstrated the ability of AGuIX to overcome resistance mechanisms to immunotherapy and highlighted the beneficial use of combining AGuIX with RT and ICIs to further improve antitumor response.

To sum up, while AGuIX alone is not sufficient to activate an immune response in T cells, NK cells or macrophages, its combination with RT is able to modulate an antitumor immune response. Indeed, AGuIX was shown to promote the release of DAMPs, including CRT and HMGB1, enhance DC maturation, reprogram TAMs, and increase CD8^+^ T cell infiltration. Furthermore, when combined with both RT and ICI anti-PD-1, AGuIX significantly increased the efficacy of both treatments, and successfully overcame resistance mechanisms to immunotherapy.

#### 5.2.5. Conclusions

All the above-mentioned results emphasize the capacity of AGuIX to trigger various types of RT-induced cell death mechanisms and to stimulate an antitumor response. Indeed, AGuIX effectively increases both early and late tumor cell apoptosis, necrosis, and autophagy. Moreover, AGuIX induces lipid peroxidation and increases the sensitivity of tumor cells to ferroptosis. Last, AGuIX triggers ICD by releasing DAMPs, which activate the immune system. The nanomedicine also directly stimulates an immune response by reprogramming TAMs into a pro-inflammatory phenotype and is able to overcome resistance to immunotherapy when combined with ICIs.

## 6. Conclusions

**Figure 9:**
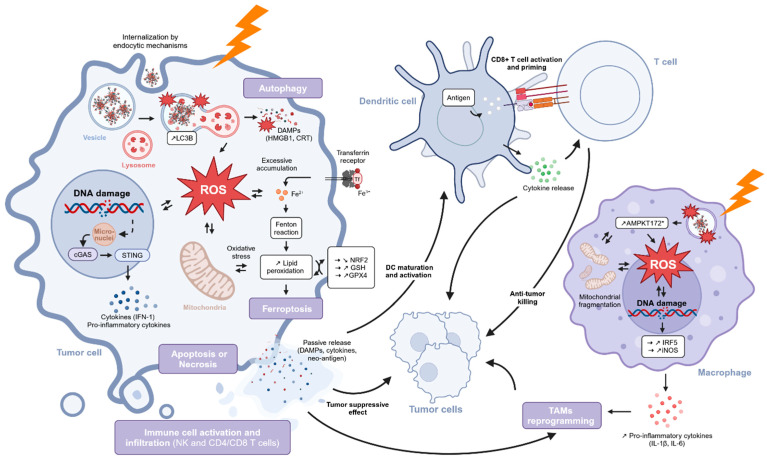
Schematic overview of the mechanisms of action of AGuIX combined with radiotherapy within the tumor microenvironment. Adenosine monophosphate activated protein kinase (AMPK) phosphorylated on threonine 172 (AMPKT172*); calreticulin (CRT); cyclic guanosine monophosphate (GMP)–AMP synthase (cGAS); damage-associated molecular patterns (DAMPs); dendritic cell (DC); glutathione peroxidase 4 (GPX4); glutathione (GSH); high-mobility group box 1 (HMGB1); interferon-1 (IFN-1); interleukin 1β (IL-1β); interleukin 6 (IL-6); inducible nitric oxide synthase (iNOS); interferon regulatory factor 5 (IRF5); microtubule-associated protein 1 light chain 3B (LC3B); nuclear factor erythroid 2-related factor 2 (NRF2); natural killer (NK); reactive oxygen species (ROS); stimulator of interferon genes (STING); tumor-associated macrophages (TAMs); transferrin (Tf). Created with BioRender.com, accessed on 11 February 2025. Note that this diagram is not to scale and is designed for improved clarity and comprehension.

AGuIX is a theranostic NP composed of gadolinium, with both positive MRI contrast agent properties and radiosensitizing potential. Its therapeutic efficacy has already been demonstrated in several types of tumors, both in vitro, in vivo and in clinical trials, highlighting its pan-cancer potential. Indeed, its mechanisms of action are not directed toward a specific biological target but based on its biodistribution and interaction with ionizing radiation and are summarized in Figure 9.

Due to its physicochemical properties, AGuIX is able to selectively accumulate in the tumor volume after its intravenous injection through the EPR effect. The NP persists in tumors for several days, and its retention properties have a major impact on its mechanisms of action. Moreover, at the cellular level, AGuIX is internalized in tumor cells by endocytic mechanisms and located in the cytoplasm, particularly in lysosomes. In parallel, the fraction of AGuIX outside the tumor volume is efficiently cleared through the kidneys within hours of administration.

In combination with EBRT, AGuIX administration is followed by one or more RT sessions that trigger numerous interdependent steps, starting from the physical interaction with matter to the chemical, biological, and immunological events that follow. Because of its composition in high-Z elements and its aggregation, AGuIX creates regions of high electron density within the tumor volume, in which a number of RT-induced events will result in greater damage to cancer cells. As demonstrated by MC simulations, AGuIX enhances the generation of secondary particles, particularly Auger electrons, leading to a localized increase in radiation dose deposition.

This step is associated with a subsequent increase in ROS production in cells, representing the chemical phase of the response to the combination of AGuIX and RT and occurring immediately after the initial physical interaction. The combined treatment also increases DNA damage, leads to the formation of more complex DNA lesions, and inhibits DNA repair ability through the HR pathway. These oxidative and genotoxic stresses induced by the combination therapy lead to specific cellular responses, such as cell cycle arrest and mitochondrial fragmentation.

Therefore, AGuIX triggers some RT-induced regulated cell death mechanisms, including apoptosis, autophagic cell death, and ferroptosis. It can activate some caspases, induce lipid peroxidation, and thereby inhibit the NRF2-GSH-GPX4 signaling pathway.

Furthermore, AGuIX combined with RT also triggers an antitumor immune response through the activation of the cGAS/STING signaling pathway and the induction of ICD with the release of DAMPS, CRT, and HMGB1. The combined treatment also modifies the TME itself by activating dendritic and T cells and reprogramming TAMs toward a pro-inflammatory phenotype that ensures tumor-suppressing functions.

To sum up, AGuIX is a nano-radiosensitizer that exhibits specific internalization and retention properties, leading to the amplification of numerous RT-induced mechanisms that are responsible for a strong therapeutic potential for many types of cancer. Beyond the phenomenon of lysosomal uptake, other mechanisms that are not yet elucidated probably occur at the cellular level, affecting tumor cells and their TME.

## 7. Discussion

Although recent preclinical and clinical results allowed for a better elucidation of the mechanisms of action of AGuIX, the current understanding is still incomplete and therefore not holistic.

First and foremost, an in-depth study to correlate macroscopic and microscopic findings regarding AGuIX biodistribution would be beneficial, as it was already observed both in MR images of patients and at the subcellular level in preclinical studies. More precisely, AGuIX biodistribution changes over time, and further optimizing the time between AGuIX administration and RT delivery is essential. Indeed, this key parameter not only conditions AGuIX safety through its elimination from healthy surrounding tissue but also its efficacy by influencing its localization in subcellular structures. Moreover, the relative percentage of circulating, extravasated, or immobilized AGuIX depends on the time elapsed between injection and irradiation.

In addition, MC simulations to date did not account for the specificity of AGuIX structure nor its subcellular localization and its eventual aggregation within these organelles. As these parameters directly influence its interaction with X-rays, they also condition the scope and magnitude of its mechanisms of action. Indeed, the advent of new computational methods that consider more complex working hypotheses, as well the biological environment, should allow progress in regard to this topic.

The chemical stage that follows the physical interaction could also be further documented, particularly in the presence of AGuIX that promotes water radiolysis. This gap includes a lack of information regarding the type of ROS produced, their quantity, their range of action, and the consequences of any interaction depending on the specific cellular organelle involved.

Beyond its effects on tumor and immune cells, interactions of AGuIX with the extracellular matrix and other components of the TME, including endothelial cells, pericytes, and fibroblasts, should be further studied. Moreover, additional studies regarding the link between AGuIX and hypoxia could be valuable, as these interactions highly depend on the oxygen levels of the cells. Beyond cellular types, the single-cell RNA sequencing (scRNA-seq) is one of the cutting-edge techniques that could help capture the diversity of cellular responses and their dynamics.

In a nutshell, a more holistic approach would complement the current state of the understanding of AGuIX’s mechanisms of action by further positioning the nanomedicine in a complex biological environment. Now that AGuIX has been quantified in several clinical studies, the next major step is to bridge preclinical and clinical results. First, correlating cellular internalization with clinical quantification by MRI would be a real asset in understanding the associated mechanisms of action. Indeed, this approach will refine and validate the therapeutic potential of AGuIX, fostering a more comprehensive understanding of its mode of action in human patients.

Regarding future research directions, published results from Phase 1 and 2 clinical trials, combined with a deeper understanding of the mechanisms of action, fuel reflections aimed at optimizing AGuIX delivery protocols for better treatment efficacy. For example, the choice of the dose, concentration, and injection rate is essential, and these parameters are relatively easy to study. On the other hand, the choice of the time between AGuIX injection and irradiation, as well as between multiple injections, is more complex to implement, especially in patients, but it is of crucial importance, nonetheless. Finally, attention must be paid to the various SOCs in RT, which sometimes vary for the same indication, including standard doses, high doses with hypofractionation, or even low doses with re-irradiation or for pediatric cancers. Understanding the mechanisms of action of a drug, although based on rather fundamental concepts, is a major translational tool in its clinical development and its optimization for improved efficacy.

## Figures and Tables

**Table 1:** Experimental conditions of in vitro studies evaluating AGuIX intracellular uptake.

Cell Line (Species and Cancer Type)	NP Incubation	Method	Reference
PANC-1 (human pancreatic adenocarcinoma)	30 min, 1, 3, 6, 24 and 48 h at 0.5 mM	MRI scanner and ICP-MS	Detappe, 2015 [66]
SQ20B (human larynx carcinoma)	1, 4, 12 and 24 h at 0.8 mM	ICP-OES	Simonet, 2020 [68]
U87 (human glioblastoma) and MCF7 (human breast adenocarcinoma)	24 h at 0.5 mg/mL	ICP-MS	Ahmad, 2020 [71]
A549 (human lung adenocarcinoma)	2 h at 0.5 mM Gd	ICP-MS	Liu, 2020 [72]
HCT-116 (human colon carcinoma; cell monolayers and spheroids)	24 h at 0.8, 1.5 and 2 mM Gd, AGuIX-Cy5.5	ICP-MS	Goodarzi, 2021 [69]
HeLa (human cervix carcinoma)	4 h at 1 mM of Gd	ICP-MS	Maury, 2021 [70]
SK-OV-3-luc (human ovarian adenocarcinoma)	18 h at 1.15 mM Gd	ICP-MS	Diaz Garcia-Prada, 2023 [50]

Magnetic resonance imaging (MRI); inductively coupled plasma–mass spectrometry (ICP-MS); inductively coupled plasma–optical emission spectrometry (ICP-OES).

**Table 2:** Experimental conditions of studies evaluating AGuIX subcellular localization.

Cell Line (Species and Cancer Type)	NP Incubation	Method and Reagent	Reference
PANC-1 (human pancreatic adenocarcinoma)	1 h at 0.5 mM	TEM	Detappe, 2015 [66]
B16F10 (mouse melanoma)	1 h at 0.6 mg/mL, AGuIX-FITC	Confocal microscopy	Kotb, 2016 [51]
	18 h at 0.6 mg/mL	TEM	
U87 (human glioblastoma)	1, 6, 16 h at 1 mM, AGuIX-Cy5.5	Confocal microscopy	Štefanciková, 2016 [67]
HepG2 (human hepatocellular carcinoma)	1 h at 0.5 mM	TEM	Hu, 2017 [55]
SQ20B (human larynx carcinoma)	24 h at 0.8 mM, AGuIX-Cy5.5	Confocal microscopy, MitoTracker green, LysoTracker green (Thermo Fisher Scientific, Saint-Aubin, France)	Simonet, 2020 [68]
H1299 (human lung carcinoma)	1 h at 1 mM, AGuIX-FITC	Inverted microscopy	Du, 2020 [48]
HCT-116 (human colon carcinoma)	24 h at 0.8, 1.5, 2 mM Gd, AGuIX-Cy5.5	Confocal microscopy, EEA1, AIF, LAMP-1 (Cell Signaling Technology, #3288, #5318, #9091).	Goodarzi, 2021 [69]
HeLa (human cervix carcinoma)	4 h at 1 mM Gd	Confocal microscopy, Cell Mask Deep Red Actin (Invitrogen)	Maury, 2021 [70]
SK-OV-3-luc (human ovarian adenocarcinoma)	18 h, AGuIX-AF488	Inverted microscopy, MitoTracker red (M7513, Thermo Fisher Scientific), LysoTracker red (L7528, Thermo Fisher Scientific)	Diaz Garcia-Prada, 2023 [50]
**Animal Model**	**NP Injection**	**Method**	**Reference**
Mice with B16F10 tumors (subcutaneous)	10 mg, intravenous	Intravital two-photon microscopy	Kotb, 2016 [51]

Transmission electron microscopy (TEM); fluorescein isothiocyanate (FITC); cyanine 5.5 (Cy5.5); early endosome antigen 1 (EEA1); apoptosis-inducing factor (AIF); lysosome-associated membrane protein 1 (LAMP-1); Alexa Fluor 488 (AF488).

**Table 3:** Experimental conditions of in vitro studies evaluating ROS production in the presence of AGuIX.

Cell Line (Species and Cancer Type)	NP Incubation	Irradiation			Fluorescence Analysis, ROS Probe	Reference
		Dose	Energy	Type		
Capan-1 (human pancreatic adenocarcinoma)	30 min at 0.43 to 1 mg/mL	4 Gy	6 MV	X-rays	Plate reader, DHR123	Detappe, 2016 [53]
SQ20B (human larynx carcinoma)	24 h at 0.8 mM	10 Gy	250 kV	X-rays	Flow cytometry, M-H_2_DCFDA or MitoSOX™ (Thermo Fisher Scientific)	Simonet, 2020 [68]
H1299 and A549 (human lung carcinoma)	1 h at 1 mM	4 Gy	662 keV	Cs^137^ γ-rays	Flow cytometry, ROS kit (DCFH-DA, Thermo Fisher Scientific, USA)	Du, 2020 [48]
B16 (mouse melanoma)	1 h at 1 mM	6 Gy	662 keV	Cs^137^ γ-rays	Flow cytometry, ROS kit (DCFH-DA, Thermo Fisher Scientific, MA, USA)	Song, 2022 [78]
MDA-MB-231 and MDA-MB-468 (human breast cancer)	1 h at 1 mM	8 Gy	662 keV	Cs^137^ γ-rays	Flow cytometry, carboxy-H_2_DCFDA (Thermo Fisher Scientific, 88-5930-74)	Sun, 2022 [77]

Dihydrorhodamine 123 (DHR123); 2′,7′-dichlorodihydrofluorescein diacetate (H_2_DCFDA or DCFH-DA); 5-(and-6)-chloromethyl-H_2_DCFDA (M-H_2_DCFDA); reactive oxygen species (ROS).

**Table 4:** Experimental conditions of in vitro studies evaluating DNA damage in the presence of AGuIX.

Cell Line (Species and Cancer Type)	NP Incubation	Irradiation			Method and Assay	Reference
		Dose	Energy	Type		
Capan-1 (human pancreatic adenocarcinoma)	15 min at 0.43 mg/mL	4 and 10 Gy	220 kVp	X-rays	IF, proportion of positive cells (>10 foci); 53BP1 (H-300, Santacruz, USA)	Detappe, 2016 [52]
Capan-1 (human pancreatic adenocarcinoma)	15 min at 0.43 mg/mL	4 Gy	6 MV	X-rays	IF, proportion of positive cells (>10 foci); 53BP1 (H-300, Santacruz, USA)	Detappe, 2016 [53]
B16F10 (mouse melanoma)	1 h at 0.6 mg/L	2 Gy	220 kV	X-rays	IF, number of foci per nucleus, γ-H2AX (Merck Millipore)	Kotb, 2016 [51]
U87 (human glioblastoma)	1, 6 and 24 h at 1 mM	1 and 4 Gy	1.173 & 1.332 MeV	^60^Co γ-rays	IF, number of foci per nucleus, γ-H2AX (Upstate Biotechnology) and 53BP1 (Cell Signaling Technology)	Štefanciková, 2016 [67]
SQ20B (human larynx carcinoma)	1 h at 0.8 mg/mL	1 Gy	75 MeV/n	^13^C^6+^ ions	IF, number of foci per nucleus, γ-H2AX	Wozny, 2017 [100]
		2 Gy	250 kV	X-rays		
MCF-7 (human breast adenocarcinoma), U87 (human glioblastoma)	24 h at 0.5 mg/mL	1 Gy	6 MV	X-rays	IF, number of foci per nucleus, 53BP1 (Novus Biologicals, USA)	Ahmad, 2020 [71]
E0771 (human breast carcinoma)	30 min at 0.4 mg/mL	2 Gy	220 kVp	X-rays	IF, number of foci per nucleus, γ-H2AX (Merck Millipore)	Detappe, 2020 [101]
H1299 and A549 (human lung carcinoma)	1 h at 1 mM	4 Gy	662 keV	Cs^137^ γ-rays	IF, proportion of positive cells (>10 foci), γ-H2AX (Merck Millipore, Belford, MA, USA) and 53BP1 (Abcam, Cambridge, UK)	Du, 2020 [48]
					AGE, olive tail moment, Comet assay	
SQ20B (human larynx carcinoma)	24 h at 0.8 mM	2 Gy	250 kV	X-rays	IF, number of foci per nucleus, γ-H2AX	Simonet, 2020 [68]
		4 Gy			AGE, tail intensity, Comet assay	
B16 (mouse melanoma)	1 h at 1 mM	6 Gy	662 keV	Cs^137^ γ-rays	IF, number of foci per nucleus, γ-H2AX	Song, 2022 [78]
MDA-MB-231 and MDA-MB-468 (human breast adenocarcinoma)	1 h at 1 mM	4 Gy	662 keV	Cs^137^ γ-rays	IF, proportion of positive cells (>10 foci), γ-H2AX (Millipore, Belford, MA, USA)	Sun, 2022 [77]
		8 Gy			AGE, olive tail moment, Comet assay	
Caco-2 (human colon adenocarcinoma)	1 h at 0.6 or 1.2 mM	6 Gy	200 keV	X-rays	IF, proportion of positive cells, γ-H2AX (Sigma, 05-636)	Tannous, 2022 [102]
Caco-2 and CT26 (murine colon carcinoma)					FM, proportion of cells with micronuclei	

Immunofluorescence (IF); agarose gel electrophoresis (AGE); fluorescence microscopy (FM). NB: Concentrations are either expressed in mg/mL when corresponding to a certain mass of AGuIX or in mM when corresponding to an amount of gadolinium element contained in AGuIX.

**Table 5:** Experimental conditions of in vivo studies evaluating DNA damage in presence of AGuIX.

Animal Model	NP Injection	Irradiation			Method and Assay	Reference
		Dose	Energy	Type		
*Nude* mice with Capan-1 tumors (subcutaneous)	250 mg/kg, 15 min prior RT, intravenous	10 Gy	220 kVp	X-rays	IHC, proportion of positive nuclei (brown), γ-H2AX (Abcam, ab26350)	Detappe, 2016 [52]
*Nude* mice with Capan-1 tumors (subcutaneous)	250 mg/kg, 15 min prior RT, intravenous	unk.	6 MV	X-rays	IHC, proportion of positive nuclei (brown), γ-H2AX (Abcam, ab11174)	Detappe, 2016 [53]
*Nude* mice with H1299 tumors (subcutaneous)	420 mg/kg, 15 min prior RT, intravenous	10 Gy	662 keV	Cs^137^ γ-rays	IHC, proportion of positive nuclei (brown), γ-H2AX (Millipore, Belford, MA, USA)	Du, 2020 [48]

Immunohistochemistry (IHC); unknown (unk., data not specified).

**Table 6:** Experimental conditions of in vitro studies evaluating cell cycle in the presence of AGuIX.

Cell Line (Species and Cancer Type)	NP Incubation	Irradiation			Method and Reagent	Reference
		Dose	Energy	Type		
H1299 (human lung carcinoma)	1 h at 1 mM	4 and 8 Gy	662 keV	Cs^137^ γ-rays	FACS, PI (Solarbio, Beijing, China)	Du, 2020 [48]
MDA-MB-231 and MDA-MB-468 (human breast adenocarcinoma)	1 h at 1 mM	4 Gy	662 keV	Cs^137^ γ-rays	FACS, PI (Solarbio, Beijing, China)	Sun, 2022 [77]
Caco-2 (human colon adenocarcinoma)	24 h at 0.6 or 1.2 mM	6 Gy	200 keV	X-rays	FACS, PI (Sigma, #P4864)	Tannous, 2022 [102]

Fluorescence-activated cell sorting (FACS); propidium iodide (PI). NB: Concentrations are expressed in mM when corresponding to an amount of gadolinium element contained in AGuIX.

**Table 7:** Experimental conditions of in vitro studies evaluating cell apoptosis and necrosis in the presence of AGuIX.

Cell Line (Species and Cancer Type)	NP Incubation	Irradiation			Method and Reagent	Reference
		Dose	Energy	Type		
Capan-1 (human pancreatic adenocarcinoma)	15 min at 0.43 mg/mL	4 Gy	220 kVp	X-rays	FACS, Annexin V-APC/7-AAD (BioLegend, USA)	Detappe, 2016 [52]
F98 (rat glioma)	6 h at 1 mM	5, 10, 20 Gy	90 keV	X-rays	FACS, Annexin V-AF488/PI (Invitrogen)	Yousef, 2016 [76]
H1299 and A549 (human lung carcinoma)	1 h at 1 mM	4 and 8 Gy	662 keV	Cs^137^ γ-ray	FACS, Annexin V-FITC (BD Pharmingen, San Diego, CA, USA)	Du, 2020 [48]
SQ20B (human larynx carcinoma)	24 h at 0.8 mM	10 Gy	250 kV	X-rays	FACS, Annexin V-FITC/PI (Life Technologies, Courtaboeuf, France) and CaspACE™ FITC-VAD-FMK marker (Promega, Charbonnières Les Bains, France)	Simonet, 2020 [68]
B16 (mouse melanoma)	1 h at 1 mM	6 Gy	662 keV	Cs^137^ γ-rays	FACS, Annexin V-FITC/PI (BD Pharmingen, San Diego, CA, USA)	Song, 2022 [78]
MDA-MB-231 and MDA-MB-468 (human breast adenocarcinoma)	1 h at 1 mM	8 Gy	662 keV	Cs^137^ γ-rays	FACS, Annexin V-FITC/PI (BD Pharmingen, San Diego, CA, USA)	Sun, 2022 [77]

Allophycocyanin (APC); 7-Amnioactinomycin D (7-AAD). NB: Concentrations are either expressed in mg/mL when corresponding to a certain mass of AGuIX or in mM when corresponding to an amount of gadolinium element contained in AGuIX.

**Table 8:** Experimental conditions of in vivo studies evaluating signs of apoptosis and necrosis in the presence of AGuIX.

Animal Model	NP Injection	Irradiation			Method and Assay	Reference
		Dose	Energy	Type		
Mice with HepG2 tumors (subcutaneous)	1 or 10 mg, intravenous, 1 h prior RT	6 Gy	300 kV	X-rays	SPECT/CT imaging; ^99m^Tc-duramycin IHC, TUNEL (Roche Diagnostics, Indianapolis, IN, USA)	Hu, 2019 [54]
*Nude* mice with H1299 tumors (subcutaneous)	420 mg/kg, intravenous, 15 min prior RT	10 Gy	662 keV	Cs^137^ γ-rays	IHC, TUNEL	Du, 2020 [48]
*Nude* mice with HEMC-SS tumors (subcutaneous)	100 mM Gd, intratumoral, 5 min prior RT	4 Gy	320 kV	X-rays	IHC, TUNEL (Promega, Madison, WI, USA)	Aloy, 2022 [49]
Mice with B16 tumors (subcutaneous)	1 mM Gd, intravenous, 4 h prior RT	4 Gy	662 keV	Cs^137^ γ-rays	IHC, TUNEL	Song, 2022 [78]
*Nude* mice with MDA-MB-231 tumors (subcutaneous)	420 mg/kg, intravenous, 30 min prior RT	10 Gy	662 keV	Cs^137^ γ-rays	IHC, TUNEL	Sun, 2022 [77]

Radiotherapy (RT); single-photon emission computed tomography/computed tomography (SPECT/CT); terminal deoxynucleotidyl transferase dUTP nick end labeling (TUNEL).

## Abbreviations

The following abbreviations are used in this manuscript:

EPR	Enhanced permeability and retention
ROS	Reactive oxygen species
RT	Radiotherapy
WHO	World Health Organization
EBRT	External beam radiation therapy
LINACs	Linear particle accelerators
TRT	Targeted radionuclide therapy
SOCs	Standards of care
LET	Linear energy transfer
BNCT	Boron neutron capture therapy
OARs	Organs at risk
TME	Tumor microenvironment
SSBs	Single-strand breaks
DSBs	Double-strand breaks
PARP	Poly-ADP ribose polymerase
NP	Nanoparticle
AGuIX	Activation and Guidance of Irradiation by X-ray
GMP	Good manufacturing practice
MRI	Magnetic resonance imaging
WBRT	Whole brain radiation therapy
SE	Signal enhancement
EES	Extracellular extravascular space
% ID	Percentage of the injected dose
SD	Standard deviation
LIBS	Laser-induced breakdown spectroscopy
ICP-MS	Inductively coupled plasma mass spectrometry
ICP-OES	Inductively coupled plasma optical emission spectrometry
TEM	Transmission electron microscopy
FITC	Fluorescein isothiocyanate
Cy5.5	Cyanine 5.5
EEA1	Early endosome antigen 1
AIF	Apoptosis-inducing factor
LAMP-1	Lysosome-associated membrane protein 1
AF488	Alexa Fluor 488
IV	Intravenous
NIST	National Institute of Standards and Technology
DOTA	1,4,7,10-tetraazacyclododecane-1,4,7,10-tetraacetic acid
LQ	Linear quadratic
SF	Survival fraction
SER	Sensitizing enhancement ratio
MC	Monte Carlo
FFF	Flattening filter free
RBE	Relative biological effectiveness
LEM	Local effect model
DEFs	Dose enhancement factors
TDRA	Theory of dual radiation action
DER	Dose enhancement ratio
DCFDA	2′,7′-dichlorofluorescein diacetate
DHR123	Dihydrorhodamine 123
H2DCFDA or DCFH-DA	2′,7′-dichlorodihydrofluorescein diacetate
M-H2DCFDA	5-(and-6)-chloromethyl-H2DCFDA
mROS	Mitochondrial ROS
cROS	Cytosolic ROS
GSH	Glutathione
SEM	Standard error of the mean
ATM	Ataxia–telangiectasia mutated
ATR	Ataxia–telangiectasia and Rad3-related protein
DDR	DNA damage response
HR	Homologous recombination
NHEJ	Non-homologous end-joining
IF	Immunofluorescence
AGE	Agarose gel electrophoresis
FM	Fluorescence microscopy
cGAS	Cyclic guanosine monophosphate–adenosine monophosphate synthase
IFN	Interferon
STING	Stimulatory interferon genes
IHC	Immunohistochemistry
Unk	Unknown
BRCA1	Breast cancer 1
FACS	Fluorescence-activated cell sorting
PI	Propidium iodide
ANOVA	Analysis of variance
APC	Allophycocyanin
7-AAD	7-Aminoactinomycin D
SPECT/CT	Single-photon emission computed tomography/computed tomography
TUNEL	Terminal deoxynucleotidyl transferase dUTP nick end labeling
H-SCORE	Histochemistry score
LC3B	Microtubule-associated protein 1 light chain 3B
BODIPY	Boron-dipyrromethene
MDA	Malondialdehyde
4-HNE	4-hydroxynonenal
NRF2	Nuclear factor erythroid 2-related factor 2
GPX4	Glutathione peroxidase 4
ns	Non-significant
ICD	Immunogenic cell death
DAMPs	Damage-associated molecular patterns
HMGB1	High-mobility group box 1
CRT	Calreticulin
DCs	Dendritic cells
TAMs	Tumor-associated macrophages
ICIs	Immune checkpoint inhibitors
PD-1	Programmed cell death protein 1
NK	Natural killer
PBMC	Peripheral blood mononuclear cells
NOX2	NADPH oxidase 2
IRF5	Interferon regulatory factor 5
iNOS	Inducible nitric oxide synthase
TOM20	Translocase of the outer mitochondrial membrane 20
AMPK	Adenosine monophosphate activated protein kinase
AMPKT172*	Activated protein kinase on threonine 172
Tregs	Regulatory T cells
MDSCs	Myeloid-derived suppressor cells
TDLNs	Tumor-draining lymph nodes
PMA	Phorbol-12-myristate 13-acetate
ROIs	Regions of interest
scRNA-seq	Single-cell RNA sequencing
IL	Interleukin
Tf	Transferrin

## References

[1] Chuang Y.C., Wu P.H., Shen Y.A., Kuo C.C., Wang W.J., Chen Y.C., Lee H.L., Chiou J.F. (2023). Recent Advances in Metal-Based NanoEnhancers for Particle Therapy. Nanomaterials.

[2] Gerken L.R.H., Gerdes M.E., Pruschy M., Herrmann I.K. (2023). Prospects of nanoparticle-based radioenhancement for radiotherapy. Mater. Horizons.

[3] Liu Y., Zhang P., Li F., Jin X., Li J., Chen W. (2018). Metal-based NanoEnhancers for Future Radiotherapy: Radiosensitizing and Synergistic Effects on Tumor Cells. Theranostics.

[4] Fazio N., Falconi M., Foglia E., Bartolomei M., Berruti A., D’Onofrio M., Ferone D., Giordano A., Grimaldi F., Milione M. (2024). Optimising Radioligand Therapy for Patients with Gastro-Entero-Pancreatic Neuroendocrine Tumours: Expert Opinion from an Italian Multidisciplinary Group. Adv. Ther..

[5] Jadvar H. (2017). Targeted Radionuclide Therapy: An Evolution Toward Precision Cancer Treatment. AJR Am. J. Roentgenol..

[6] Vozenin M.C., Bourhis J., Durante M. (2022). Towards clinical translation of FLASH radiotherapy. Nat. Rev. Clin. Oncol..

[7] Malouff T.D., Seneviratne D.S., Ebner D.K., Stross W.C., Waddle M.R., Trifiletti D.M., Krishnan S. (2021). Boron Neutron Capture Therapy: A Review of Clinical Applications. Front. Oncol..

[8] Cho B. (2018). Intensity-modulated radiation therapy: A review with a physics perspective. Radiat. Oncol. J..

[9] Mijnheer B., Beddar S., Izewska J., Reft C. (2013). In vivo dosimetry in external beam radiotherapy. Med. Phys..

[10] Jaffray D.A. (2012). Image-guided radiotherapy: From current concept to future perspectives. Nat. Rev. Clin. Oncol..

[11] Brock K.K. (2019). Adaptive Radiotherapy: Moving Into the Future. Semin. Radiat. Oncol..

[12] Noël G., Antoni D. (2022). Organs at risk radiation dose constraints. Cancer Radiother..

[13] Boustani J., Grapin M., Laurent P.A., Apetoh L., Mirjolet C. (2019). The 6th R of Radiobiology: Reactivation of Anti-Tumor Immune Response. Cancers.

[14] Ward J.F. (1988). DNA damage produced by ionizing radiation in mammalian cells: Identities, mechanisms of formation, and reparability. Prog. Nucleic Acid Res. Mol. Biol..

[15] Baskar R., Dai J., Wenlong N., Yeo R., Yeoh K.W. (2014). Biological response of cancer cells to radiation treatment. Front. Mol. Biosci..

[16] Small K.L., Henthorn N.T., Angal-Kalinin D., Chadwick A.L., Santina E., Aitkenhead A., Kirkby K.J., Smith R.J., Surman M., Jones J. (2021). Evaluating very high energy electron RBE from nanodosimetric pBR322 plasmid DNA damage. Sci. Rep..

[17] Azzam E.I., Jay-Gerin J.P., Pain D. (2012). Ionizing radiation-induced metabolic oxidative stress and prolonged cell injury. Cancer Lett..

[18] Gong L., Zhang Y., Liu C., Zhang M., Han S. (2021). Application of Radiosensitizers in Cancer Radiotherapy. Int. J. Nanomedicine.

[19] Pernin V., Mégnin-Chanet F., Pennaneach V., Fourquet A., Kirova Y., Hall J. (2014). PARP inhibitors and radiotherapy: Rational and prospects for a clinical use. Cancer Radiother..

[20] Sun C., Chu A., Song R., Liu S., Chai T., Wang X., Liu Z. (2023). PARP inhibitors combined with radiotherapy: Are we ready?. Front. Pharmacol..

[21] Li M.H., Ito D., Sanada M., Odani T., Hatori M., Iwase M., Nagumo M. (2004). Effect of 5-fluorouracil on G1 phase cell cycle regulation in oral cancer cell lines. Oral Oncol..

[22] Crane C.H., Skibber J.M., Birnbaum E.H., Feig B.W., Singh A.K., Delclos M.E., Lin E.H., Fleshman J.W., Thames H.D., Kodner I.J. (2003). The addition of continuous infusion 5-FU to preoperative radiation therapy increases tumor response, leading to increased sphincter preservation in locally advanced rectal cancer. Int. J. Radiat. Oncol. Biol. Phys..

[23] Romani A.M.P. (2022). Cisplatin in cancer treatment. Biochem. Pharmacol..

[24] Douple E.B., Richmond R.C., O’Hara J.A., Coughlin C.T. (1985). Carboplatin as a potentiator of radiation therapy. Cancer Treat. Rev..

[25] Famurewa A.C., Mukherjee A.G., Wanjari U.R., Sukumar A., Murali R., Renu K., Vellingiri B., Dey A., Valsala Gopalakrishnan A. (2022). Repurposing FDA-approved drugs against the toxicity of platinum-based anticancer drugs. Life Sci..

[26] Cartwright B.M., Corso J.N., Lightner J., Whitted C., Torrenegra R.D., Krishnan K., Palau V.E. (2023). Achyrocline B (3,5 dihydroxy-6,7,8-trimethoxyflavone) synergizes with 5-fluorouracil allowing for dose reduction and reduced off-target toxicity in the treatment of colonic and pancreatic cancers. Biomed. Pharmacother..

[27] McMahon S.J., Hyland W.B., Muir M.F., Coulter J.A., Jain S., Butterworth K.T., Schettino G., Dickson G.R., Hounsell A.R., O’Sullivan J.M. (2011). Biological consequences of nanoscale energy deposition near irradiated heavy atom nanoparticles. Sci. Rep..

[28] Penninckx S., Heuskin A.C., Michiels C., Lucas S. (2020). Gold Nanoparticles as a Potent Radiosensitizer: A Transdisciplinary Approach from Physics to Patient. Cancers.

[29] Penninckx S., Martinive P., Mirjolet C. (2023). Radiation-activated nanoparticles: Which combination to optimize radiosensitization?. Cancer/Radiothérapie.

[30] Hainfeld J.F., Slatkin D.N., Smilowitz H.M. (2004). The use of gold nanoparticles to enhance radiotherapy in mice. Phys. Med. Biol..

[31] Zhang R., Kiessling F., Lammers T., Pallares R.M. (2023). Clinical translation of gold nanoparticles. Drug Deliv. Transl. Res..

[32] Bulin A.L., Broekgaarden M., Chaput F., Baisamy V., Garrevoet J., Busser B., Brueckner D., Youssef A., Ravanat J.L., Dujardin C. (2020). Radiation Dose-Enhancement Is a Potent Radiotherapeutic Effect of Rare-Earth Composite Nanoscintillators in Preclinical Models of Glioblastoma. Adv. Sci..

[33] Aubrun Fulbert C., Chaput F., Stelse-Masson S., Henry M., Chovelon B., Bohic S., Brueckner D., Garrevoet J., Moriscot C., Gallet B. (2024). Nanoscintillator Coating: A Key Parameter That Strongly Impacts Internalization, Biocompatibility, and Therapeutic Efficacy in Pancreatic Cancer Models. Small Sci..

[34] Le Duc G., Miladi I., Alric C., Mowat P., Bräuer-Krisch E., Bouchet A., Khalil E., Billotey C., Janier M., Lux F. (2011). Toward an image-guided microbeam radiation therapy using gadolinium-based nanoparticles. ACS Nano.

[35] Lux F., Tran V.L., Thomas E., Dufort S. (2018). AGuIX ^®^ from bench to bedside—Transfer of an ultrasmall theranostic gadolinium-based nanoparticle to clinical medicine. Br. J. Radiol..

[36] Zhang P., Marill J., Darmon A., Anesary N.M., Lu B., Paris S. (2021). NBTXR3 Radiotherapy-Activated Functionalized Hafnium Oxide Nanoparticles Show Efficient Antitumor Effects Across a Large Panel of Human Cancer Models. Int. J. Nanomed..

[37] Li Y., Yun K.H., Lee H., Goh S.H., Suh Y.G., Choi Y. (2019). Porous platinum nanoparticles as a high-Z and oxygen generating nanozyme for enhanced radiotherapy in vivo. Biomaterials.

[38] Deng J., Xu S., Hu W., Xun X., Zheng L., Su M. (2018). Tumor targeted, stealthy and degradable bismuth nanoparticles for enhanced X-ray radiation therapy of breast cancer. Biomaterials.

[39] Scher N., Bonvalot S., Le Tourneau C., Chajon E., Verry C., Thariat J., Calugaru V. (2020). Review of clinical applications of radiation-enhancing nanoparticles. Biotechnol. Rep..

[40] Bonvalot S., Le Pechoux C., De Baere T., Kantor G., Buy X., Stoeckle E., Terrier P., Sargos P., Coindre J.M., Lassau N. (2017). First-in-Human Study Testing a New Radioenhancer Using Nanoparticles (NBTXR3) Activated by Radiation Therapy in Patients with Locally Advanced Soft Tissue Sarcomas. Clin. Cancer Res..

[41] Bonvalot S., Rutkowski P.L., Thariat J., Carrère S., Ducassou A., Sunyach M.P., Agoston P., Hong A., Mervoyer A., Rastrelli M. (2019). NBTXR3, a first-in-class radioenhancer hafnium oxide nanoparticle, plus radiotherapy versus radiotherapy alone in patients with locally advanced soft-tissue sarcoma (Act.In.Sarc): A multicentre, phase 2-3, randomised, controlled trial. Lancet Oncol..

[42] Mignot A., Truillet C., Lux F., Sancey L., Louis C., Denat F., Boschetti F., Bocher L., Gloter A., Stéphan O. (2013). A Top-Down Synthesis Route to Ultrasmall Multifunctional Gd-Based Silica Nanoparticles for Theranostic Applications. Chem.–A Eur. J..

[43] Rocchi P. (2022). Conception de Nanoparticules de Seconde Génération Issues d’ AGuIX ^®^ Pour une Application en Oncologie. Ph.D. Thesis.

[44] Matsumura Y., Maeda H. (1986). A New Concept for Macromolecular Therapeutics in Cancer Chemotherapy: Mechanism of Tumoritropic Accumulation of Proteins and the Antitumor Agent Smancs. Cancer Res..

[45] Verry C., Dufort S., Lemasson B., Grand S., Pietras J., Troprès I., Crémillieux Y., Lux F., Mériaux S., Larrat B. (2020). Targeting brain metastases with ultrasmall theranostic nanoparticles, a first-in-human trial from an MRI perspective. Sci. Adv..

[46] Verry C., Dufort S., Villa J., Gavard M., Iriart C., Grand S., Charles J., Chovelon B., Cracowski J., Quesada J. (2021). Theranostic AGuIX nanoparticles as radiosensitizer: A phase I, dose-escalation study in patients with multiple brain metastases (NANO-RAD trial). Radiother. Oncol..

[47] Chargari C., Maury P., Texier M., Genestie C., Morice P., Bockel S., Gouy S., Ba M., Achkar S., Lux F. (2024). Theragnostic gadolinium-based nanoparticles safely augment X-ray radiation effects in patients with cervical cancer. ACS Nano.

[48] Du Y., Sun H., Lux F., Xie Y., Du L., Xu C., Zhang H., He N., Wang J., Liu Y. (2020). Radiosensitization Effect of AGuIX, a Gadolinium-Based Nanoparticle, in Nonsmall Cell Lung Cancer. ACS Appl. Mater. Interfaces.

[49] Aloy M.T., Boumedine J.S., Deville A., Kryza D., Gauthier A., Brichart-Vernos D., Ollier G., La Padula V., Lux F., Tillement O. (2022). Proof of Concept of the Radiosensitizing Effect of Gadolinium Oxide Nanoparticles in Cell Spheroids and a Tumor-Implanted Murine Model of Chondrosarcoma. Int. J. Nanomed..

[50] Diaz Garcia-Prada C., Carmes L., Atis S., Parach A., Bertolet A., Jarlier M., Poty S., Garcia D.S., Shin W.G., Du Manoir S. (2023). Gadolinium-Based Nanoparticles Sensitize Ovarian Peritoneal Carcinomatosis to Targeted Radionuclide Therapy. J. Nucl. Med..

[51] Kotb S., Detappe A., Lux F., Appaix F., Barbier E.L., Tran V.L., Plissonneau M., Gehan H., Lefranc F., Rodriguez-Lafrasse C. (2016). Gadolinium-based nanoparticles and radiation therapy for multiple brain melanoma metastases: Proof of concept before phase I trial. Theranostics.

[52] Detappe A., Kunjachan S., Sancey L., Motto-Ros V., Biancur D., Drane P., Guieze R., Makrigiorgos G.M., Tillement O., Langer R. (2016). Advanced multimodal nanoparticles delay tumor progression with clinical radiation therapy. J. Control. Release.

[53] Detappe A., Kunjachan S., Drané P., Kotb S., Myronakis M., Biancur D.E., Ireland T., Wagar M., Lux F., Tillement O. (2016). Key clinical beam parameters for nanoparticle-mediated radiation dose amplification. Sci. Rep..

[54] Hu P., Fu Z., Liu G., Tan H., Xiao J., Shi H., Cheng D. (2019). Gadolinium-Based Nanoparticles for Theranostic MRI-Guided Radiosensitization in Hepatocellular Carcinoma. Front. Bioeng. Biotechnol..

[55] Hu P., Cheng D., Huang T., Banizs A.B., Xiao J., Liu G., Chen Q., Wang Y., He J., Shi H. (2017). Evaluation of Novel 64Cu-Labeled Theranostic Gadolinium-Based Nanoprobes in HepG2 Tumor-Bearing Nude Mice. Nanoscale Res. Lett..

[56] Dufort S., Le Duc G., Salomé M., Bentivegna V., Sancey L., Bräuer-Krisch E., Requardt H., Lux F., Coll J.L., Perriat P. (2016). The High Radiosensitizing Efficiency of a Trace of Gadolinium-Based Nanoparticles in Tumors. Sci. Rep..

[57] Sancey L., Kotb S., Truillet C., Appaix F., Marais A., Thomas E., Van Der Sanden B., Klein J.P., Laurent B., Cottier M. (2015). Long-term in Vivo clearance of gadolinium-based AGuIX nanoparticles and their biocompatibility after systemic injection. ACS Nano.

[58] Bennett S., Verry C., Kaza E., Miao X., Dufort S., Boux F., Crémillieux Y., de Beaumont O., Le Duc G., Berbeco R. (2024). Quantifying gadolinium-based nanoparticle uptake distributions in brain metastases via magnetic resonance imaging. Sci. Rep..

[59] Biau J., Durando X., Boux F., Molnar I., Moreau J., Leyrat B., Guillemin F., Lavielle A., Cremillieux Y., Seddik K. (2024). NANO-GBM trial of AGuIX nanoparticles with radiotherapy and temozolomide in the treatment of newly diagnosed Glioblastoma: Phase 1b outcomes and MRI-based biodistribution. Clin. Transl. Radiat. Oncol..

[60] Le Duc G., Roux S., Paruta-Tuarez A., Dufort S., Brauer E., Marais A., Truillet C., Sancey L., Perriat P., Lux F. (2014). Advantages of gadolinium based ultrasmall nanoparticles vs molecular gadolinium chelates for radiotherapy guided by MRI for glioma treatment. Cancer Nanotechnol..

[61] Tillement O., Lux F., Dufort S., Verry C., LeDuc G. (2021). Methods for Treating Tumors. WO 2021/019268. https://hal.science/hal-04753043.

[62] Zarschler K., Rocks L., Licciardello N., Boselli L., Polo E., Garcia K.P., De Cola L., Stephan H., Dawson K.A. (2016). Ultrasmall inorganic nanoparticles: State-of-the-art and perspectives for biomedical applications. Nanomed. Nanotechnol. Biol. Med..

[63] Epple M., Rotello V.M., Dawson K. (2023). The Why and How of Ultrasmall Nanoparticles. Acc. Chem. Res..

[64] Verry C., Sancey L., Dufort S., Le Duc G., Mendoza C., Lux F., Grand S., Arnaud J., Quesada J.L., Villa J. (2019). Treatment of multiple brain metastases using gadolinium nanoparticles and radiotherapy: NANO-RAD, a phase I study protocol. BMJ Open.

[65] Tofts P.S., Brix G., Buckley D.L., Evelhoch J.L., Henderson E., Knopp M.V., Larsson H.B.W., Lee T.-Y., Mayr N.A., Parker G.J.M. (1999). Estimating kinetic parameters from dynamic contrast-enhanced t1-weighted MRI of a diffusable tracer: Standardized quantities and symbols. J. Magn. Reson. Imaging.

[66] Detappe A., Kunjachan S., Rottmann J., Robar J., Tsiamas P., Korideck H., Tillement O., Berbeco R. (2015). AGuIX nanoparticles as a promising platform for image-guided radiation therapy. Cancer Nanotechnol..

[67] Štefanciková L., Lacombe S., Salado D., Porcel E., Pagáčová E., Tillement O., Lux F., Depeš D., Kozubek S., Falk M. (2016). Effect of gadolinium-based nanoparticles on nuclear DNA damage and repair in glioblastoma tumor cells. J. Nanobiotechnol..

[68] Simonet S., Rodriguez-Lafrasse C., Beal D., Gerbaud S., Malesys C., Tillement O., Lux F., Fayyad-Kazan H., Rachidi W., Ardail D. (2020). Gadolinium-Based Nanoparticles Can Overcome the Radioresistance of Head and Neck Squamous Cell Carcinoma Through the Induction of Autophagy. J. Biomed. Nanotechnol..

[69] Goodarzi S., Prunet A., Rossetti F., Bort G., Tillement O., Porcel E., Lacombe S., Wu T.-D., Guerquin-Kern J.L., Delanoë-Ayari H. (2021). Quantifying nanotherapeutic penetration using a hydrogel-based microsystem as a new 3D in vitro platform. Lab Chip.

[70] Maury P., Porcel E., Mau A., Lux F., Tillement O., Mahou P., Schanne-Klein M.C., Lacombe S. (2021). Rapid Evaluation of Novel Therapeutic Strategies Using a 3D Collagen-Based Tissue-Like Model. Front. Bioeng. Biotechnol..

[71] Ahmad R., Schettino G., Royle G., Barry M., Pankhurst Q.A., Tillement O., Russell B., Ricketts K. (2020). Radiobiological Implications of Nanoparticles Following Radiation Treatment. Part. Part. Syst. Charact..

[72] Liu W., Deacon J., Yan H., Sun B., Liu Y., Hegan D., Li Q., Coman D., Parent M., Hyder F. (2020). Tumor-targeted pH-low insertion peptide delivery of theranostic gadolinium nanoparticles for image-guided nanoparticle-enhanced radiation therapy. Transl. Oncol..

[73] Varzandeh M., Labbaf S., Varshosaz J., Laurent S. (2022). An overview of the intracellular localization of high-Z nanoradiosensitizers. Prog. Biophys. Mol. Biol..

[74] Štefančíková L., Porcel E., Eustache P., Li S., Salado D., Marco S., Guerquin-Kern J.L., Réfrégiers M., Tillement O., Lux F. (2014). Cell localisation of gadolinium-based nanoparticles and related radiosensitising efficacy in glioblastoma cells. Cancer Nanotechnol..

[75] Rima W., Sancey L., Aloy M.T., Armandy E., Alcantara G.B., Epicier T., Malchère A., Joly-Pottuz L., Mowat P., Lux F. (2013). Internalization pathways into cancer cells of gadolinium-based radiosensitizing nanoparticles. Biomaterials.

[76] Yousef I., Seksek O., Gil S., Prezado Y., Sulé-Suso J., Martínez-Rovira I. (2016). Study of the biochemical effects induced by X-ray irradiations in combination with gadolinium nanoparticles in F98 glioma cells: First FTIR studies at the Emira laboratory of the SESAME synchrotron. Analyst.

[77] Sun H., Cai H., Xu C., Zhai H., Lux F., Xie Y., Feng L., Du L., Liu Y., Sun X. (2022). AGuIX nanoparticles enhance ionizing radiation-induced ferroptosis on tumor cells by targeting the NRF2-GPX4 signaling pathway. J. Nanobiotechnol..

[78] Song H., Sun H., He N., Xu C., Wang Y., Du L., Liu Y., Wang Q., Ji K., Wang J. (2022). Gadolinium-based ultra-small nanoparticles augment radiotherapy-induced T-cell response to synergize with checkpoint blockade immunotherapy. Nanoscale.

[79] Carter J.D., Cheng N.N., Qu Y., Suarez G.D., Guo T. (2007). Nanoscale energy deposition by X-ray absorbing nanostructures. J. Phys. Chem. B.

[80] Yan H., Carlson D.J., Abolfath R., Liu W. (2021). Microdosimetric investigation and a novel model of radiosensitization in the presence of metallic nanoparticles. Pharmaceutics.

[81] Mott J.H.L., Daniel J.M. (2021). Interactions of Electromagnetic Radiation and Subatomic Particles with Matter—Part 1. Clin. Oncol..

[82] McMahon S.J., Paganetti H., Prise K.M. (2016). Optimising element choice for nanoparticle radiosensitisers. Nanoscale.

[83] Lux F., Detappe A., Dufort S., Sancey L., Louis C., Carme S., Tillement O. (2015). Ultrasmall nanoparticles for radiotherapy: AGuIX. Cancer/Radiotherapie.

[84] Wu J., Xu X., Liang Y., Chen T., Quan E., Wang L. (2023). Biological modeling of gadolinium-based nanoparticles radio-enhancement for kilovoltage photons: A Monte Carlo study. Cancer Nanotechnol..

[85] Ge Y., Ji X., Zhang R., Li K., Chen G.H. (2017). K-edge energy-based calibration method for photon counting detectors. Phys. Med. Biol..

[86] Maury P., Mondini M., Chargari C., Darricau A., Shahin M., Ammari S., Bockel S., Genestie C., Wu T.-D., Lux F. (2023). Clinical transfer of AGuIX^®^-based radiation treatments for locally advanced cervical cancer: MR quantification and in vitro insights in the NANOCOL clinical trial framework. Nanomed. Nanotechnol. Biol. Med..

[87] Delorme R., Taupin F., Flaender M., Ravanat J.L., Champion C., Agelou M., Elleaume H. (2017). Comparison of gadolinium nanoparticles and molecular contrast agents for radiation therapy-enhancement. Med. Phys..

[88] Cho S.H. (2005). Estimation of tumour dose enhancement due to gold nanoparticles during typical radiation treatments: A preliminary Monte Carlo study. Phys. Med. Biol..

[89] Mesbahi A., Jamali F., Gharehaghaji N. (2013). Effect of photon beam energy, gold nanoparticle size and concentration on the dose enhancement in radiation therapy. BioImpacts.

[90] Subiel A., Ashmore R., Schettino G. (2016). Standards and Methodologies for Characterizing Radiobiological Impact of High-Z Nanoparticles. Theranostics.

[91] Hernández Millares R., Bae C., Kim S.J., Kim T., Park S.Y., Lee K., Ye S.J. (2024). Clonogenic assay and computational modeling using real cell images to study physical enhancement and cellular sensitization induced by metal nanoparticles under MV and kV X-ray irradiation. Nanoscale.

[92] Jia S., Ge S., Fan X., Leong K.W., Ruan J. (2021). Promoting reactive oxygen species generation: A key strategy in nanosensitizer-mediated radiotherapy. Nanomedicine.

[93] Howard D., Sebastian S., Le Q.V.C., Thierry B., Kempson I. (2020). Chemical mechanisms of nanoparticle radiosensitization and radioprotection: A review of structure-function relationships influencing reactive oxygen species. Int. J. Mol. Sci..

[94] Torii S., Shintoku R., Kubota C., Yaegashi M., Torii R., Sasaki M., Suzuki T., Mori M., Yoshimoto Y., Takeuchi T. (2016). An essential role for functional lysosomes in ferroptosis of cancer cells. Biochem. J..

[95] Nakamura H., Takada K. (2021). Reactive oxygen species in cancer: Current findings and future directions. Cancer Sci..

[96] Sia J., Szmyd R., Hau E., Gee H.E. (2020). Molecular Mechanisms of Radiation-Induced Cancer Cell Death: A Primer. Front. Cell Dev. Biol..

[97] Brandsma I., Gent D.C. (2012). Pathway choice in DNA double strand break repair: Observations of a balancing act. Genome Integr..

[98] Baatout S. (2023). Radiobiology Textbook.

[99] Choi J., Kim G., Bin Cho S., Im H.-J. (2020). Radiosensitizing high-Z metal nanoparticles for enhanced radiotherapy of glioblastoma multiforme. J. Nanobiotechnol..

[100] Wozny A.S., Aloy M.T., Alphonse G., Magné N., Janier M., Tillement O., Lux F., Beuve M., Rodriguez-Lafrasse C. (2017). Gadolinium-based nanoparticles as sensitizing agents to carbon ions in head and neck tumor cells. Nanomed. Nanotechnol. Biol. Med..

[101] Detappe A., Mathieu C., Jin C., Agius M.P., Diringer M.C., Tran V.L., Pivot X., Lux F., Tillement O., Kufe D. (2020). Anti-MUC1-C Antibody–Conjugated Nanoparticles Potentiate the Efficacy of Fractionated Radiation Therapy. Int. J. Radiat. Oncol. Biol. Phys..

[102] Tannous D. (2022). The Combination of Gadolinium-Based Nanoparticles, Radiotherapy, and Immune Checkpoint Inhibitors: A Novel Therapeutic Opportunity for Cancer Treatment. PhD Thesis.

[103] Elmore S. (2007). Apoptosis: A Review of Programmed Cell Death. Toxicol. Pathol..

[104] Wu Y.H., Chen R.J., Chiu H.W., Yang L.X., Wang Y.L., Chen Y.Y., Yeh Y.L., Liao M.Y., Wang Y.J. (2023). Nanoparticles augment the therapeutic window of RT and immunotherapy for treating cancers: Pivotal role of autophagy. Theranostics.

[105] Wen J., Zhang X. (2023). HMGB1 Signaling-Mediated Tumor Immunity in Cancer Progress. Front. Biosci..

[106] Zhang M., Xiao J., Liu J., Bai X., Zeng X., Zhang Z., Liu F. (2022). Calreticulin as a marker and therapeutic target for cancer. Clin. Exp. Med..

[107] Diamond J.M., Vanpouille-Box C., Spada S., Rudqvist N.-P., Chapman J.R., Ueberheide B.M., Pilones K.A., Sarfraz Y., Formenti S.C., Demaria S. (2018). Exosomes Shuttle TREX1-Sensitive IFN-Stimulatory dsDNA from Irradiated Cancer Cells to DCs. Cancer Immunol. Res..

[108] Wu Q., Allouch A., Paoletti A., Leteur C., Mirjolet C., Martins I., Voisin L., Law F., Dakhli H., Mintet E. (2017). NOX2-dependent ATM kinase activation dictates pro-inflammatory macrophage phenotype and improves effectiveness to radiation therapy. Cell Death Differ..

[109] Craig D.J., Nanavaty N.S., Devanaboyina M., Stanbery L., Hamouda D., Edelman G., Dworkin L., Nemunaitis J.J. (2021). The Abscopal Effect of Radiation Therapy. Future Oncol..

[110] Yi M., Zheng X., Niu M., Zhu S., Ge H., Wu K. (2022). Combination strategies with PD-1/PD-L1 blockade: Current advances and future directions. Mol. Cancer.

[111] Penninckx S., Thariat J., Mirjolet C. (2023). Radiation therapy-activated nanoparticle and immunotherapy: The next milestone in oncology?. Int. Rev. Cell Mol. Biol..

[112] Muradova Z., Tannous D., Mostefa-Kara A., Cao-Pham T.T., Lamy C., Broutin S., Paci A., Dufort S., Doussineau T., Lux F. (2024). Gadolinium-based nanoparticles AGuIX and their combination with ionizing radiation trigger AMPK-dependent proinflammatory reprogramming of tumor-associated macrophages. bioRxiv.

[113] Mittelheisser V., Lefebvre O., Banerjee M., Ghosh S., Dupas A., Diringer M.-C., Blumberger J., Bochler L., Harlepp S., Larnicol A. (2024). Nanomaterials trigger functional responses in primary human immune cells. bioRxiv.

